# Flexible Embedded Metal Meshes by Sputter-Free Crack Lithography for Transparent Electrodes and Electromagnetic Interference Shielding

**DOI:** 10.1021/acsami.3c16405

**Published:** 2024-01-27

**Authors:** Mehdi Zarei, Mingxuan Li, Elizabeth E. Medvedeva, Sooraj Sharma, Jungtaek Kim, Zefan Shao, S. Brett Walker, Melbs LeMieux, Qihan Liu, Paul W. Leu

**Affiliations:** † Department of Mechanical Engineering, University of Pittsburgh, Pittsburgh, Pennsylvania 15261, United States; ‡ Department of Chemical Engineering, University of Pittsburgh, Pittsburgh, Pennsylvania 15261, United States; § Department of Bioengineering, University of Pittsburgh, Pittsburgh, Pennsylvania 15261, United States; ∥ Department of Materials Science, University of Pittsburgh, Pittsburgh, Pennsylvania 15261, United States; ⊥ Department of Industrial Engineering, University of Pittsburgh, Pittsburgh, Pennsylvania 15261, United States; # Electroninks Incorporated, Austin, Texas 78744, United States

**Keywords:** EMI shielding, metal ink, reactive-ion etching, crack lithography, metal mesh, flexible transparent electrode

## Abstract

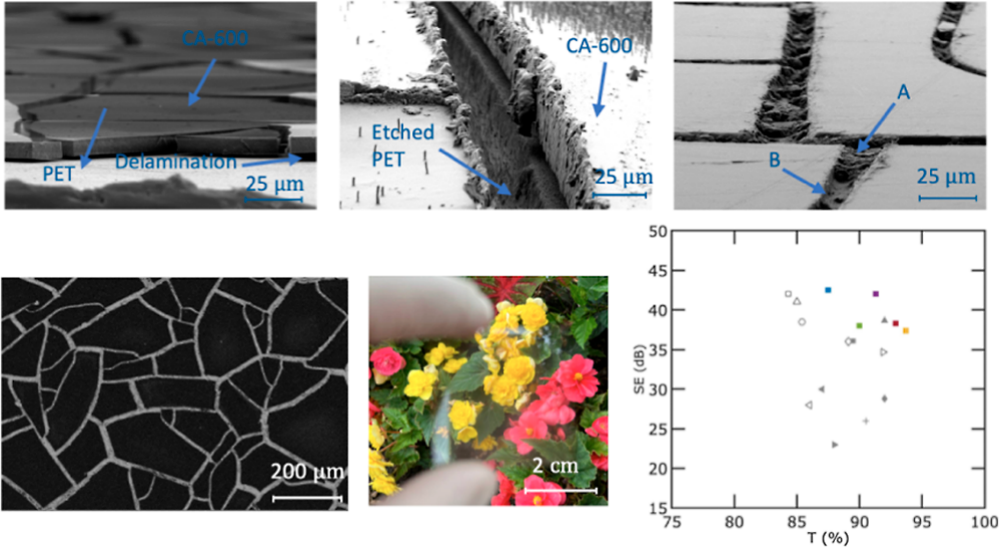

A facile and novel fabrication method is demonstrated for creating flexible poly(ethylene terephthalate) (PET)-embedded silver meshes using crack lithography, reactive ion etching (RIE), and reactive silver ink. The crack width and spacing in a waterborne acrylic emulsion polymer are controlled by the thickness of the polymer and the applied stress due to heating and evaporation. Our innovative fabrication technique eliminates the need for sputtering and ensures stronger adhesion of the metal meshes to the PET substrate. Crack trench depths over 5 μm and line widths under 5 μm have been achieved. As a transparent electrode, our flexible embedded Ag meshes exhibit a visible transmission of 91.3% and sheet resistance of 0.54 Ω/sq as well as 93.7% and 1.4 Ω/sq. This performance corresponds to figures of merit (σ_DC_/σ_OP_) of 7500 and 4070, respectively. For transparent electromagnetic interference (EMI) shielding, the metal meshes achieve a shielding efficiency (SE) of 42 dB with 91.3% visible transmission and an EMI SE of 37.4 dB with 93.7% visible transmission. We demonstrate the highest transparent electrode performance of crack lithography approaches in the literature and the highest flexible transparent EMI shielding performance of all fabrication approaches in the literature. These metal meshes may have applications in transparent electrodes, EMI shielding, solar cells, and organic light-emitting diodes.

## Introduction

Radio frequency (RF) electromagnetic radiation has revolutionized communication networks. As we become more reliant on electronic devices and systems, there is a pressing need for the development of effective electromagnetic interference (EMI) shielding materials. These shielding materials protect electronic components from radiation damage and block undesirable signals.^1−7^ To address EMI shielding needs, a wide variety of materials have been studied including metal films,^1,3,8−11^ metal meshes,^12−16^ metal nanowires,^17−19^ graphene,^20^ carbon nanotubes,^21,22^ and MXenes.^23^ There is also growing interest in hybrid structures,^24−26^ such as conductive oxide/metal,^27−29^ MXene/metal,^30,31^ and metal/graphene.^32,33^

Furthermore, many modern optoelectronic devices, such as light-emitting diodes, car windows, displays, touchscreens, solar cells, and optical communication systems, need materials that are both visibly transparent and capable of blocking RF signals.^34^ However, achieving this balance is challenging. Some materials, such as MXenes, thin film materials, graphene, metal nanowires, and carbon nanotubes, lose their effectiveness when transparency is needed. To maintain transparency without sacrificing too much shielding efficiency (SE),^28,35−37^ these materials are often combined with conductive polymers like poly(3,4-ethylenedioxythiophene) polystyrene sulfonate (PEDOT/PSS)^32,33^ or high-refractive-index materials like indium tin oxide (ITO) or zinc oxide (ZnO). Our previous research explored the capabilities of metal thin film/metal oxide combinations in this context.^35^

Metal networks, such as metal meshes and metal nanowires, have been the subject of much research for transparent conducting electrodes with high transparency and low sheet resistance.^38−41^ Therefore, there has been considerable interest in adapting these materials for applications in transparent EMI shielding. Metal nanowires have challenges due to their nonuniform distribution, inherent percolation limitations, and significant contact resistance between wires. In contrast, metal meshes tend to maintain consistent uniform properties without percolation or contact issues. Several well-established methodologies have been developed for the fabrication of metal meshes, including photolithography,^29,42−50^ imprint lithography,^51−53^ 3D printing,^54,55^ crack lithography,^32,56−62^ electrodeposition,^29,63^ e-beam direct writing,^64^ and self-assembly.^65^ Most of these methods, however, are costly, time-intensive, and often unsuitable for large-scale production. Specifically, imprint lithography, which uses a prefabricated mold to imprint patterns onto a substrate, struggles with mold removal, ensuring proper contact, and fabricating complex designs. This method also requires different molds for varying widths and pitches to manage the balance between transparency (T) and SE.^51^ Photolithography, another common method, creates uniform, small-width patterns. However, its application may not be well-suited for large-scale applications due to its reliance on costly equipment and cleanroom conditions. The process is further complicated by the use of physical vapor deposition of metallic film and the lift-off technique.

Crack lithography has been the focus of much attention as a relatively straightforward approach. This method leverages cracks that form in a thin film when the accumulated internal stress exceeds a certain limit. This stress can arise from several external sources, including thermal, mechanical, and environmental factors.^66^ Fabricating a metal mesh using crack lithography typically involves five main steps: preparing a colloidal solution or gel film, applying it to the substrate, allowing cracks to form due to induced stresses, depositing a metallic film, and finally removing the crack template. Common materials used for crack template films include acrylic resin,^67,68^ SiO_2_ or TiO_2_ nanoparticle-based dispersions,^32,69^ poly(methyl methacrylate),^70^ and egg white.^58,71^

Most studies in this field have utilized sputtering to form a conductive metallic film. However, a significant drawback of this technique, especially when working with narrow, high-aspect-ratio cracks, is the risk of the lift-off process inadvertently removing the deposited metal.^72^ Additionally, conductive metals such as silver (Ag) and copper (Cu) tend to adhere poorly to substrate surfaces, which typically necessitates the use of a costly titanium adhesive layer.^73^ An alternative to sputtering is electrodeposition of the metallic films.^71,74^ However, this method requires either a seed layer or a conductive substrate (commonly ITO/glass).^29^ Using a rigid, conductive substrate can impede the application of the formed metals for flexible optoelectronics. While electrodeposition can enhance sheet resistance, it may also reduce transmission because the process often increases metal line widths.

In this study, we provide a novel approach in the field of crack lithography focusing on the creation of flexible embedded metal meshes. The effects of the curing temperature and crack template thickness were investigated on the width and spacing of the cracks. Our facile fabrication method involves transferring the crack patterns to a PET substrate using reactive-ion etching (RIE), which enables the precise transfer of narrow-width patterns to the substrate with a high aspect ratio. The process also eliminates the need for the sputtering process, traditionally employed in crack lithography for producing conductive meshes. After removing the crack template, we fill the embedded cracks with a highly conductive Ag ink and cure the ink at a low temperature below 110 °C. This method not only streamlines the fabrication process but also enhances the adhesion strength of the resulting mesh.

Our flexible embedded Ag meshes achieve 91.3% visible transmission and a sheet resistance (*R*_s_) of 0.54, which correspond to a σ_DC_/σ_OP_ of 7500. These meshes also achieve 93.7% visible transmission and *R*_s_ = 1.40 Ω/sq, which correspond to σ_DC_/σ_OP_ = 4070. For transparent EMI shielding, the samples exhibit an SE of 42 dB with 91.3% visible transmission and an SE of 37.4 with 93.7% visible transmission. Our results outperform other crack lithography works in the literature in both transparent electrode and EMI shielding performance. These metal meshes achieve the best transparent EMI shielding performance due to the combination of high-conductivity silver, large thickness of mesh (over 5 μm), and small line widths (under 5 μm). Embedded structures exhibit greater robustness during bending tests compared with that of sputtered metallic films on the surface. The integration of metal meshes into PET offers various advantages for different flexible applications including transparent electrodes, solar cells, organic light-emitting diodes, and optoelectronic devices.

## Results and Discussion

**Figure 1:**
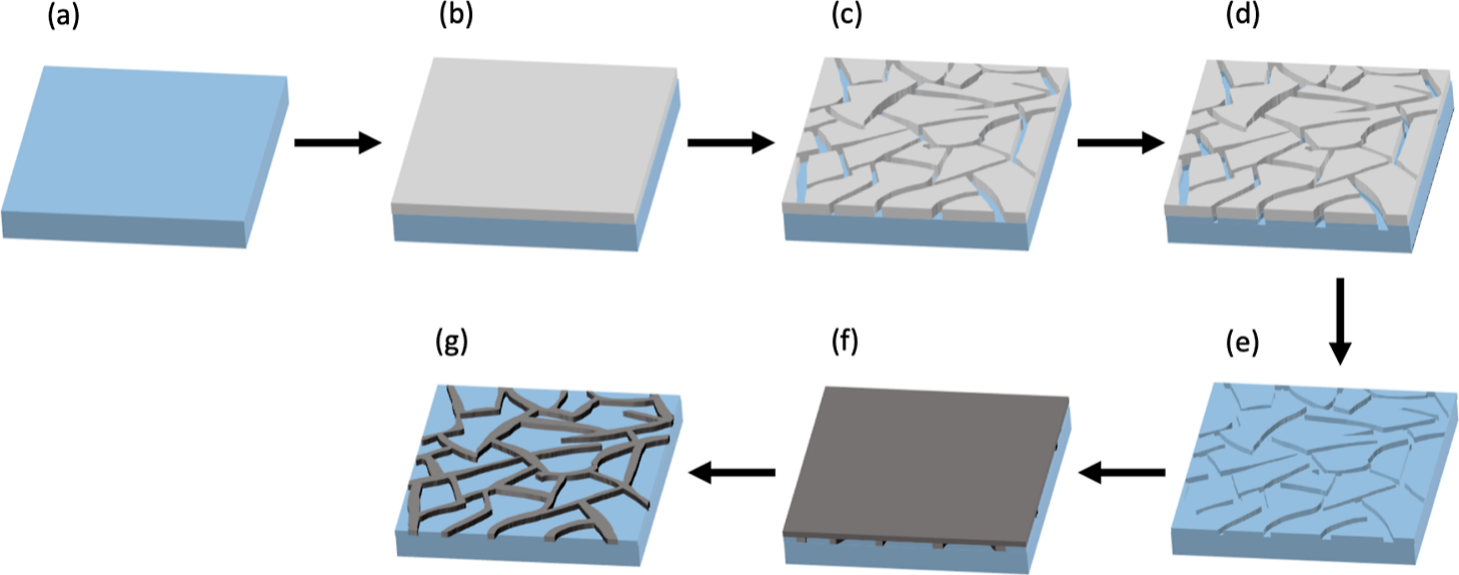
Schematic of flexible PET-embedded Ag mesh fabrication process: (a) transparent flexible PET substrate, (b) crack template film spin-coating, (c) formation of cracks due to heating, (d) reactive ion etching, (e) crack template film removal, (f) Ag ink coating and curing, and finally (g) removal of Ag by a wiper and final hard curing.

Figure 1 shows the schematic of the fabrication process. A transparent flexible PET substrate (Figure 1a) was spin-coated with CA-600 as a crack template (Figure 1b). Carboset CA-600 is a commercially available waterborne acrylic emulsion polymer. Then, as shown in Figure 1c, during the curing process, induced stress in the polymer leads to crack formation. The cracks were transferred to the PET substrate using RIE (Figure 1d). The crack template film was removed through ultrasonication in acetone (Figure 1e). Silver ink was drop-cast onto the PET and ramp-cured (Figure 1f). The excess ink was removed after a soft curing step, followed by hard curing of the ink (Figure 1g). The film thickness and consequently the crack patterns can be manipulated through the spin coating speed and curing temperature, as will be discussed later. We transferred the self-forming cracks to the PET using RIE with a gas flow of 50 sccm CF_4_ and 20 sccm SF_6_, a pressure of 30 mT, and a power of 250 W. RIE is well-suited for etching high-aspect-ratio anisotropic structures, such as deep trenches or narrow channels. This capability is essential for creating complex patterns with varying depths.^42,75^ RIE provides control over the etch rate, resulting in varying trench thicknesses.^42,43^ However, it is important to note that the crack template layer is also susceptible to etching. This imposes a limitation on the maximum etching time and, consequently, the achievable depth of the trenches. Overetching could lead to increased surface roughness in the areas in between the cracks. These defects are potential locations for trapping silver ink during the curing process, which results in lower transmission and worse performance. This fabrication process may also be adapted for rigid substrates such as glass, though the etch chemistry will be different and the achievable depth of trenches is likely to be less, as glass is generally harder and more resistant to etching compared to PET. Following the etching process, Ag ink is coated onto the PET by drop casting and then cured. The ink is initially soft-cured at temperatures ramping up from 70 to 110 °C. Excess Ag is removed from the sample using a cleanroom wipe. The Ag ink is then hard-cured at 110 °C, ensuring that the silver adheres firmly and securely to the PET substrate.

Table 1 provides details on the process parameters and structures of the nine samples fabricated for our experiments. The process parameters include the spin speed, the curing temperature of the CA-600, and the RIE etch time. The structure parameters include the crack template thickness, the trench depth, as well as the line width and spacing of the cracks. The thicknesses of the crack template were measured through side-view scanning electron microscopy (SEM) images. The depth of the etched trenches was determined from optical profilometry. Figure S1 shows the depth of the etched cracks in the five studied samples, determined by optical profilometry measurements for the three different etch times of 950, 750, and 650 s. These samples have uniform crack depths of 5.9, 5.3, and 5.0 μm, respectively. Thicker crack template films enable larger depth cracks as the substrate can be etched for a longer time before the crack template is etched through. We are interested in investigating the processing factors that affect the width, spacing, and thickness of the cracks as these dimensions play a critical role in determining the performance of the samples in terms of transmission and EMI shielding.

**Table 1:** Summary of the Fabrication and Structural Parameters for Various Metal Mesh Samples Fabricated

sample	spin speed (rpm)	curing temp. (°C)	CA-600 thickness (μm)	crack width (μm)	crack spacing (μm)	etch time (s)	trench depth (μm)
1	1000	60	9.2	12.1 ± 3.5	156.9 ± 54.7	950	5.9
2	1500	60	6.8	5.4 ± 1.2	120.8 ± 35.8	750	5.3
3	2000	60	5.9	3.8 ± 0.9	101.9 ± 36.6	650	5.0
4	1000	80	9.2	8.1 ± 1.6	176.4 ± 56.7	950	5.9
5	1500	80	6.8	4.2 ± 1.1	132.6 ± 48.9	750	5.3
6	2000	80	5.9				
7	1000	100	9.2				
8	1500	100	6.8				
9	2000	100	5.9				

**Figure 2:**
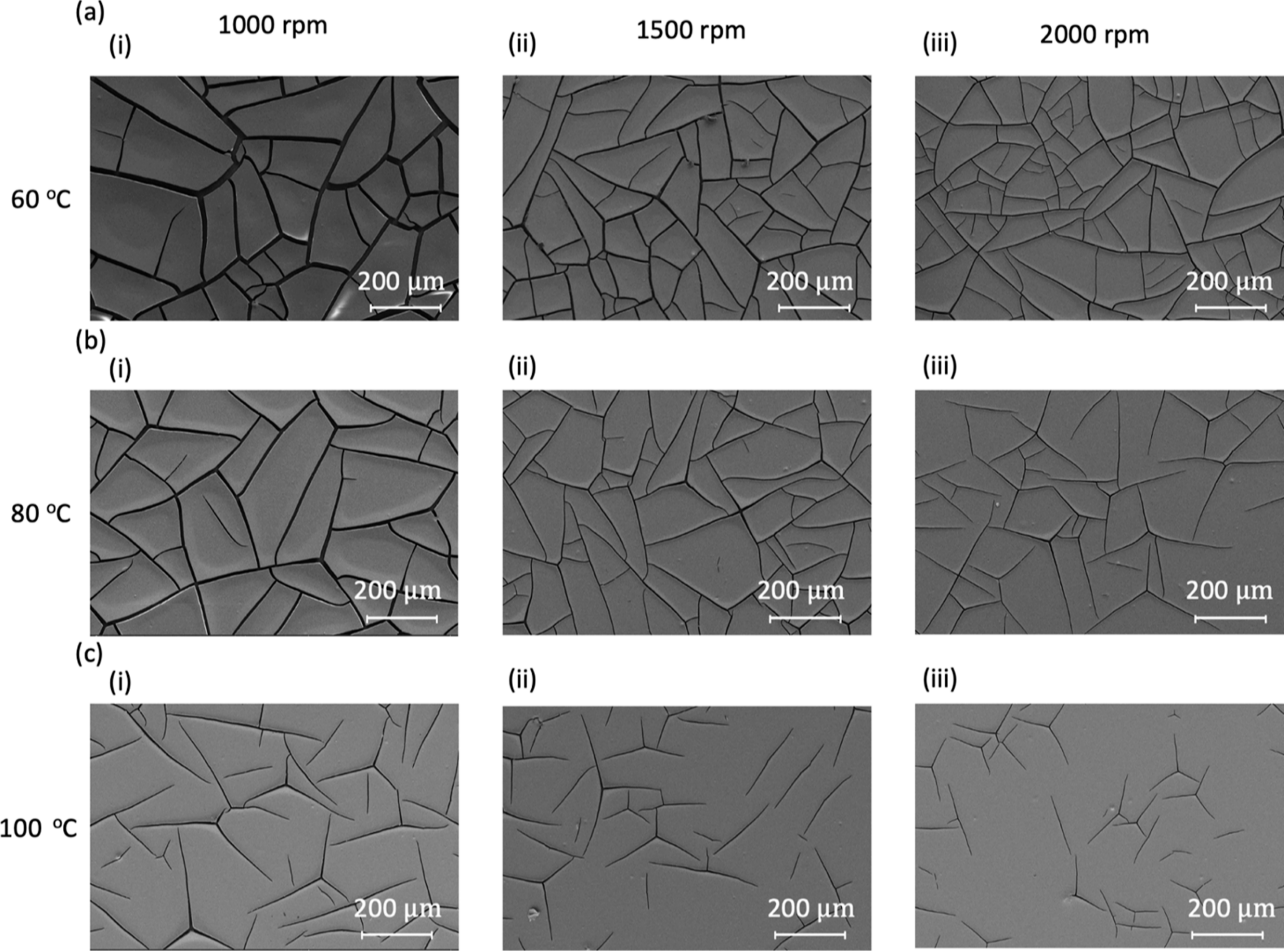
SEM images of nine samples showing the effects of different curing temperatures and spin speeds on crack formation. The curing temperatures are (a) 60, (b) 80, and (c) 100 °C. The spin speeds are (i) 1000, (ii) 1500, and (iii) 2000 rpm. Of the nine samples, five exhibit cracks forming isolated domains in the polymer, which will be used for creating metal meshes. These samples are (a)(i), (a)(ii), (a)(iii), (b)(i), and (b)(ii).

Figure 2 provides SEM images illustrating the effects of spin speed and curing temperature on crack formation for nine different samples. The CA-600 is cured at different temperatures of (a) 60, (b) 80, and (c) 100 °C and varying spin speeds of (i) 1000, (ii) 1500, and (iii) 2000 rpm. Five of the nine fabricated samples feature cracks which create isolated domains in the polymer film, which can be used to create metal meshes. These samples are (a)(i), (a)(ii), (a)(iii), (b)(i), and (b)(ii). For these five samples, we used image analysis to identify the centroids of each domain, as depicted in Figure S2. Domains that appear cropped at the edges of the SEM images are excluded from our calculations to ensure accuracy. The spacing of the cracks is then determined from the area of the domains. We also measured and examined the distribution of crack widths and spacing in the five samples. Mean crack widths as small as 1.8 μm were observed in sample 3. The width distribution measurements for the five samples are shown in Figure S3. Summary statistics on the crack spacing and crack widths with the average and standard deviation are listed in Table 1.

The process of film cracking due to drying, known as desiccation-induced film cracking, has been previously studied.^69,76,77^ As the film dries, it experiences tensile stress, leading to crack formation. The likelihood of cracking is determined by the film’s tensile stress; specifically, thicker and more brittle films are more prone to cracking.^69,76^ The critical condition for film cracking is
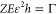
1where *E* is the Young’s modulus of the film, ε represents the drying-induced mismatch strain between the film and the substrate, *h* is the film thickness, and *Z* is a numerical factor related to the crack mode (for the channeling crack observed in our study, *Z* = 1.976).^78^ Γ, the fracture toughness of the film, opposes cracking.

Experimental results confirm that increased spinning rates and higher curing temperatures lead to reduced cracking. Our observations show that higher spinning speeds during film application result in thinner cracks, decreasing the driving force for cracking. Additionally, a higher curing temperature promotes faster film creep, reducing the mismatch strain ε. This creep behavior is particularly sensitive to temperature as our curing occurs near the film’s glass transition temperature of 50 °C. Although both the Young’s modulus *E* and fracture toughness Γ vary with temperature, after curing and returning to room temperature, only ε impacts the equation.

The stochastic cracking process is influenced by the initial distribution of nucleation sites and the dynamics of the crack interaction. As the film dries, the mismatch strain ε increases, which drives the nucleation, propagation, and widening of the cracks. The thickness of the film is a key factor in this process; as the thickness increases, so do the width and spacing of the cracks. When the drying temperature is higher, the mismatch strain ε is reduced, which lessens the force, causing the cracks to widen or new cracks to form. As a result, the cracks in films dried at higher temperatures tend to be narrower and further apart. This pattern is consistent with previous studies linking film thickness, crack spacing, and width.^76^ Our data align with these theoretical predictions, showing wider average crack widths and spacing with thicker films and narrower spacing with lower curing temperatures. Furthermore, samples with thicker films and lower curing temperatures have a wider range of crack widths. This variation is due to earlier crack initiation in the drying process, leading to different growth durations and final outcomes.

**Figure 3:**
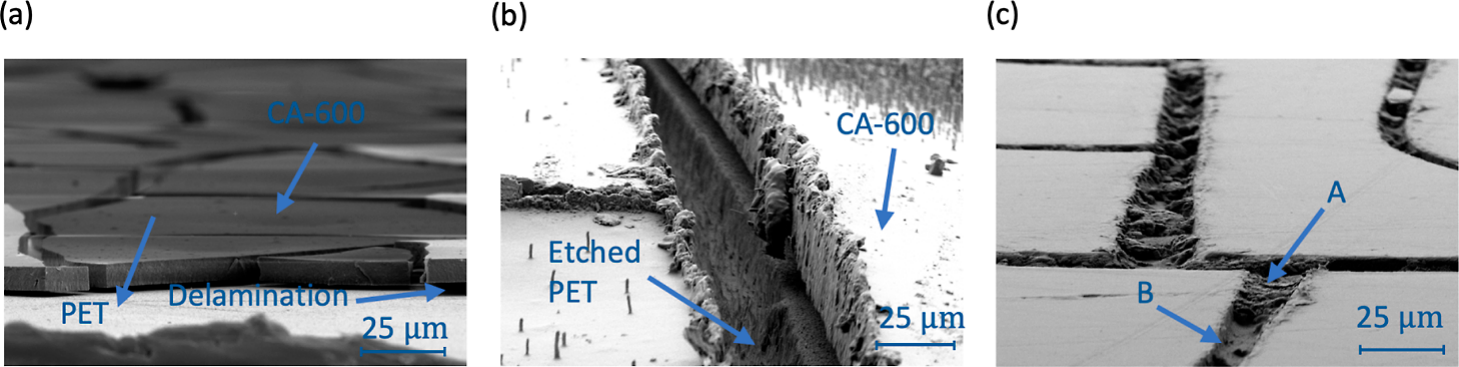
SEM images showing crack formation at different stages of fabrication processes: (a) crack formation after curing at an 88° angle from directly overhead, (b) transferring cracks into the PET through reactive ion etching at an 83° angle from directly overhead, and (c) after removal of crack template and Ag filling at an 85° angle from directly overhead.

Consequently, we fabricated samples 1–5 into PET-embedded metal meshes. Samples 6–9 were not processed into metal meshes as the cracks were not interconnected. Figure 3 displays SEM images illustrating the progression of crack formation at various stages of the fabrication process. In Figure 3a, we observe the initial formation of cracks postcuring on a PET substrate, identifiable by its white hue underneath. A noteworthy observation is the prevalent delamination of the crack template from the PET substrate due to insufficient adhesion. While such delamination has been problematic in other methodologies, particularly sputtering and lift-off processes, it does not impede our process since we transfer the patterns directly into the PET and then remove the sacrificial layer. It is important to note that attempting to apply metal ink prior to transferring the cracks to the PET is impractical as the ink can seep under the delaminated cells, compromising the substrate’s transparency. Thus, transferring the cracks into the PET is a crucial step, allowing us to use metal ink instead of sputtering.

In Figure 3b, the cracks have been successfully transferred to the PET substrate through reactive ion etching, creating parallel embedded crack walls in the PET. The etching process also etches the crack template film, which, if allowed to progress, can ultimately damage the PET substrate in regions between cracks, forming increased roughness and small holes. This roughness and holes can trap silver ink during the curing process, negatively impacting transparency. Consequently, the etching time is a critical parameter, directly related to the thickness of the template film. A thicker template can endure longer etching, resulting in deeper trenches. After etching, the samples were subjected to ultrasonication in acetone to completely remove the template CA-600 film prior to filling the trenches with Ag ink. In Figure 3c, we observe the Ag-filled embedded metal meshes in the SEM images at an 85° angle from the overhead perspective. The absence of silver in noncrack regions indicates successful removal of excess silver during the wiping process. Despite this, there are areas of under-filling (B) contrasted with well-filled areas (A), suggesting that adjusting the trench thickness could enhance the filling uniformity. We observe that narrower trenches tend to fill more uniformly than wider ones. This phenomenon occurs due to a weak edge-pinning effect so that some of the ink extends above the trench edges, forming a concave meniscus due to the effects of surface tension. This concave meniscus aids in achieving a more thorough and uniform fill of the trenches during the curing process. Additionally, maintaining the flatness of the PET substrate during these processes is crucial to ensure that ink does not spread beyond the edges of the substrate. This is important to prevent contamination of the back side and the hot plate underneath as such contamination can lead to a failure in the curing process.

**Figure 4:**
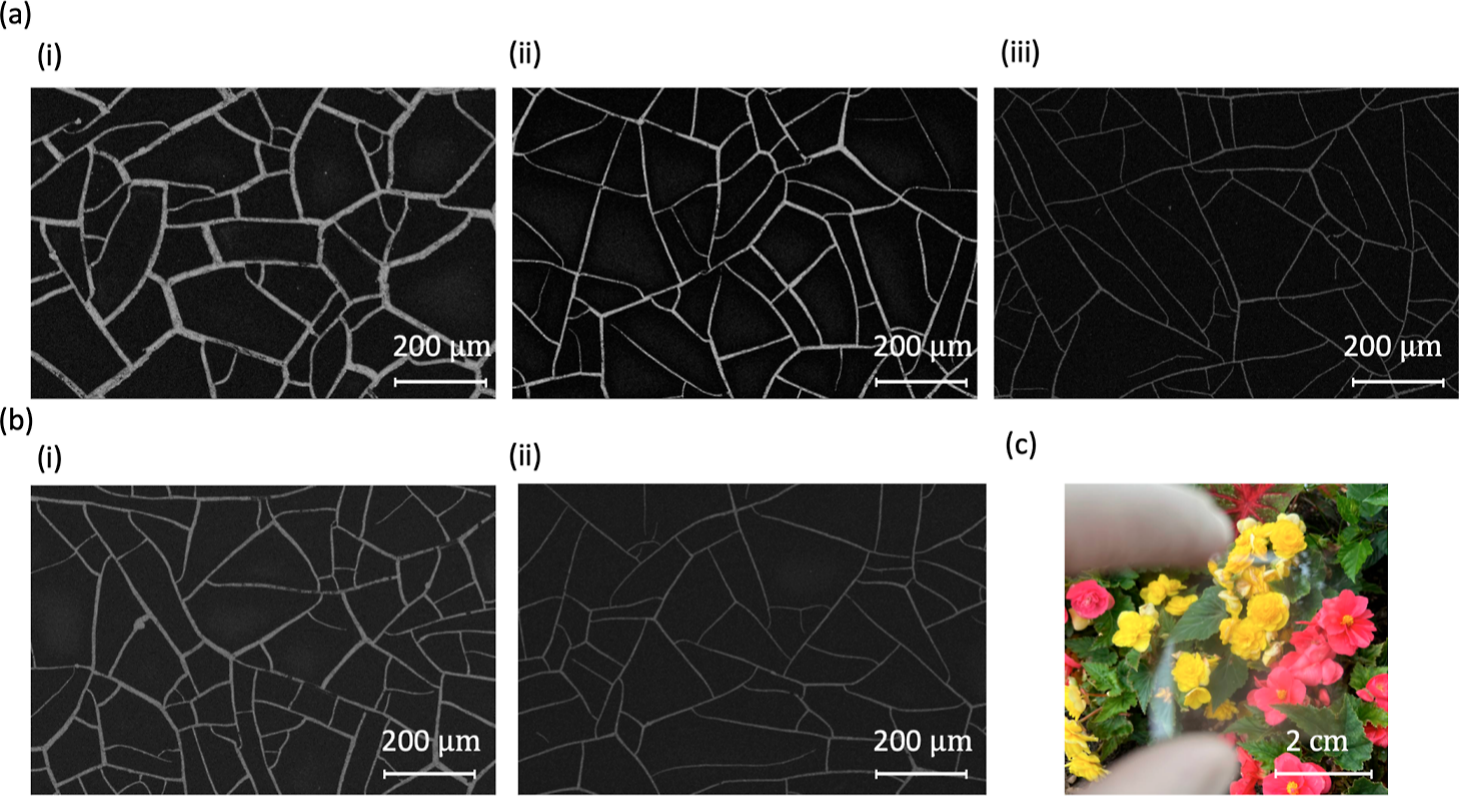
Top-view SEM images displaying Ag filling of crack-embedded patterns into the PET of samples 1–5, respectively, with interconnected cracks for two curing temperatures: (a) 60 and (b) 80 °C, as well as varying spin speeds: (i) 1000, (ii) 1500, and (iii) 2000. (c) Optical image of sample 4 with a *T* = 90.2% and haze = 18.0% at 550 nm.

Figure 4a,b shows the top-view SEM images of the five fabricated metal meshes, revealing that the trenches are uniformly and thoroughly filled with silver, and importantly, no silver residue is present between the trenches. This level of uniformity is crucial, particularly for optoelectronic applications.^42^ The trend of decreasing the crack width from left to right and top to bottom is also evident in the SEM images. This observation indicates that RIE is capable of accurately replicating the crack width into PET, without enlarging or reducing the widths. This can be attributed to the capability of RIE to execute anisotropic etching, indicating a directional etching process. Sample 6, which had a crack template prepared at 80 °C and spin speed of 2000 rpm, along with samples 7 through 9, all processed at 100 °C, was not fabricated into metal meshes. This was due to the poorly interconnected cracks observed in those samples, making them unsuitable for the creation of metal meshes. Figure 4c displays an optical image of sample 4 with *T* = 90.2% at 550 nm. The image clearly demonstrates the sample’s optical clarity and high transmission in the visible spectrum.

**Figure 5:**
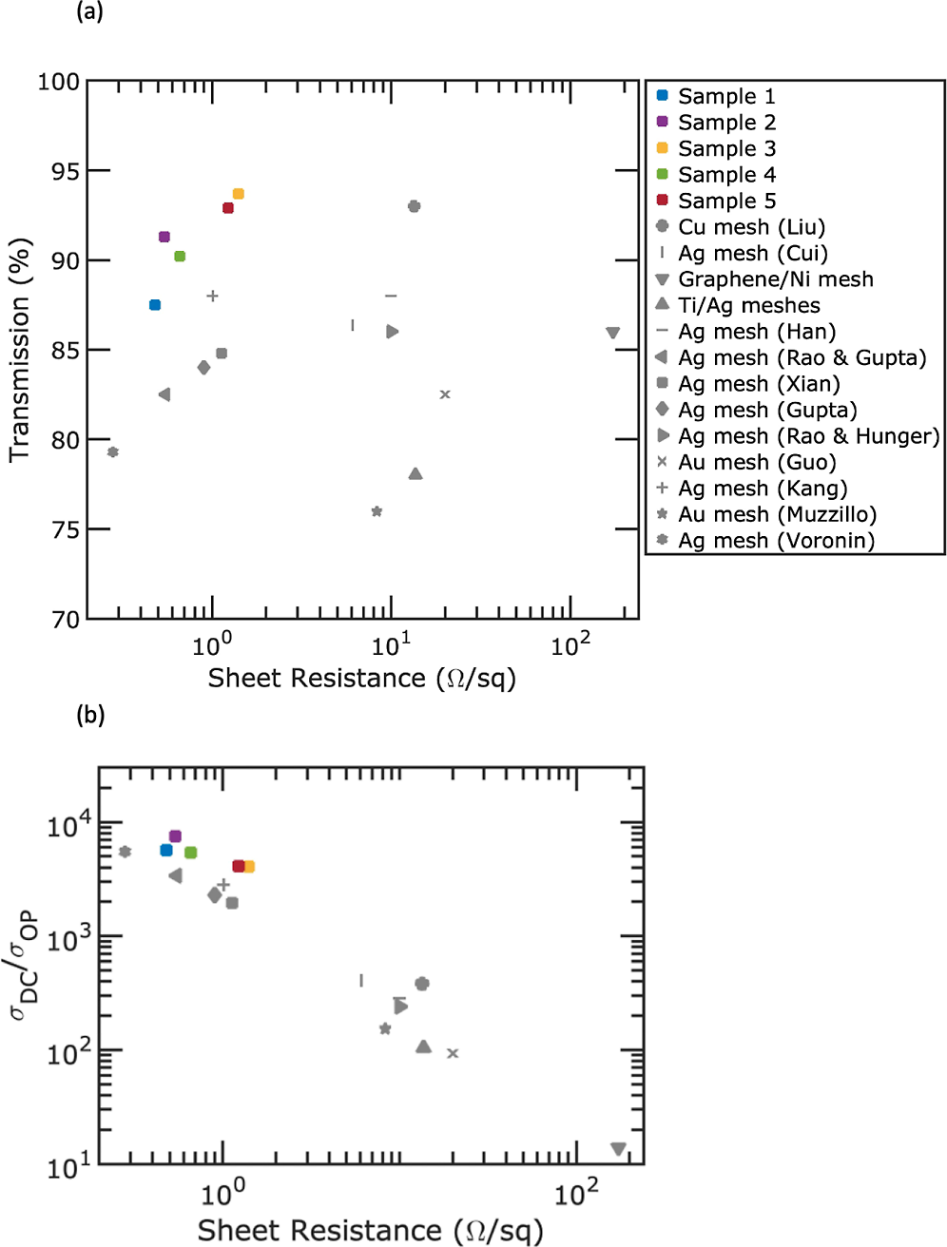
Transparent electrode performance of PET-embedded Ag meshes for our five samples compared to that of other crack lithography metal meshes in the literature. (a) Transmission at 550 nm versus sheet resistance and (b) σ_DC_/σ_OP_ versus sheet resistance. The following works in the literature are shown: Cu mesh(Liu),^67^ Ag mesh (Cui),^68^ graphene/Ni mesh,^32^ Ti/Ag meshes,^73^ Ag mesh (Han),^69^ Ag mesh (Rao and Gupta),^76^ Ag mesh (Xian),^71^ Ag mesh (Gupta),^79^ Ag mesh (Rao and Hunger),^80^ Au mesh (Guo),^81^ Ag mesh (Kang),^74^ Au mesh (Muzzillo),^70^ and Ag mesh (Voronin).^58^

Figure 5 presents the transparent electrode performance of our embedded Ag mesh samples. Figure 5a plots the transmission at 550 nm wavelength and *R*_s_ for five samples we fabricated compared to that of other crack lithography transparent electrodes in the literature, including Cu mesh (Liu),^67^ Ag mesh (Cui),^68^ graphene/Ni mesh,^32^ Ti/Ag meshes,^73^ Ag mesh (Han),^69^ Ag mesh (Rao and Gupta),^76^ Ag mesh (Xian),^71^ Ag mesh (Gupta),^79^ Ag mesh (Rao and Hunger),^80^ Au mesh (Guo),^81^ Ag mesh (Kang),^74^ Au mesh (Muzzillo),^70^ and Ag mesh (Voronin).^58^ Our samples 1 through 5 demonstrate transmissions of 87.5, 91.3, 93.7, 90.2, and 92.9% with corresponding *R*_s_ of 0.48, 0.54, 1.4, 0.66, and 1.23 (Ω/sq), respectively. Figure 5b shows σ_DC_/σ_OP_ for various metal meshes as a function of *R*_s_. The figure of merit is calculated by the following
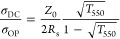
2where *Z*_0_ = 377 Ω is the free space impedance and *T*_550_ is the transparency at 550 nm wavelength. This metric is the ratio between their direct current conductivity (σ_DC_) and their optical conductivity (σ_OP_). A higher figure of merit signifies a more efficient device in transmitting greater electrical current while preserving excellent optical transparency. Our samples 1 through 5 achieve figures of merit of 5690, 7500, 4070, 5400, and 4090, respectively.

Our samples demonstrate superior performance as transparent electrodes compared to that of other crack lithography studies, primarily by overcoming the limitations associated with sputtering processes. Since sputtering of metal meshes and the subsequent lift-off processes have limitations in achieving thick metal meshes, lower conductance hinders their widespread applications.^72^ A comprehensive performance comparison of our metal meshes with other crack lithography transparent electrodes in the literature is provided in Table S1. Electroplating and electrodeposition methods have been used to increase the thickness of metal meshes after the crack template lift-off, but these methods have their limitations as metal tends to deposit isotropically, which can lead to an undesirable increase in mesh width.^74^

**Figure 6:**
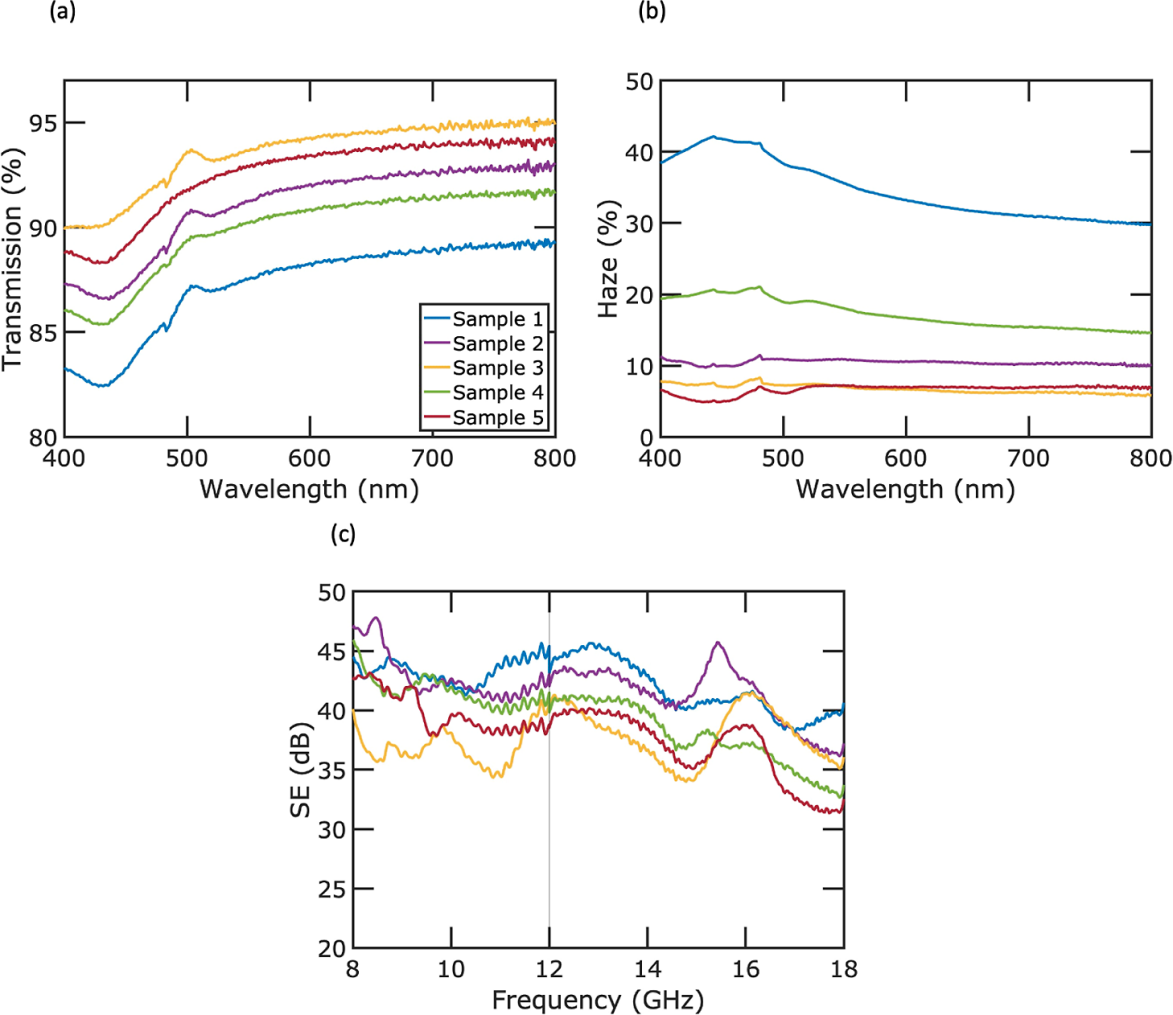
Optical and SE performance of five Ag mesh samples. (a) Transmission versus wavelength within the visible spectrum, (b) haze versus wavelength within the visible spectrum, and (c) EMI SE vs frequency in the range of 8–18 GHz.

Figure 6a presents the optical transmission results of our Ag mesh samples across the visible spectrum, ranging from 400 to 800 nm wavelength. The transmission values were adjusted to exclude the influence of the substrate. Specifically, we calculated these adjusted transmission values by dividing the measured transmission of the samples by the transmission of the substrate alone. The transmittance of light through silver increases as the wavelength gets longer. This is because silver exhibits a surface plasmon resonance around 326 nm, which leads to a higher absorption and reduced transmission at lower wavelengths. Figure 6b presents haze measurements on all of the samples within the visible spectrum. Haze refers to the percentage of total transmitted light that is scattered. Our findings show that haze values for the samples range from 7.0 to 36.3%. Specifically, samples with narrower line widths of 3.8 and 4.2 μm exhibited lower haze values (7.0 and 7.2%, respectively), whereas the sample with a line width of 12.1 μm had a significantly higher haze of 36.3%. These results suggest that modifying the spin speed and curing temperature during crack formation allows for effective control over haze. The ability to achieve a wide range of haze values is beneficial for optoelectronic applications as some require low haze (like displays, windows, filters, and lenses) and others benefit from high haze (such as lighting and solar cells).

Figure 6c shows the EMI SE versus frequency in the range of 8–18 GHz. The SE is calculated by
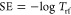
3where *T*_rf_ denotes the radio frequency transparency from 8 to 18 GHz. We evaluated the SE across two specific frequency bands: the X-band (8–12 GHz), utilized in radar systems for air traffic control, weather monitoring, and military applications, and the Ku-band (12 to 18 GHz), primarily employed in satellite communications. Our five fabricated metal meshes achieved average SE values of 42.5, 42.0, 37.4, 39.7, and 38.3 dB, respectively, across the specified frequency range. Notably, the SE of all samples shows little variation within this range, indicating consistent and effective EMI shielding. Such performance is important in military and aerospace applications.

Figure S4 provides detailed insights into the SE performance of the metal meshes. The total SE is a combination of the reflection shielding efficiency SE_R_ and absorption efficiency SE_A_. For the five sample tests, the reflection efficiencies are 13.1, 13.0, 14.0, 13.3, and 13.1 dB, respectively, while the absorption efficiencies are notably higher at 29.4, 29.0, 23.4, 26.4, and 25.2 dB, respectively. Additionally, Figure S5b provides the power coefficients for transmission, reflection, and absorption at radio frequencies for these five samples. The reflection coefficients *R*_rf_ are consistently high across the samples, at 0.95, 0.95, 0.96, 0.95, and 0.95. In comparison, the absorption coefficients *A*_rf_ are lower, at 0.05, 0.05, 0.04, 0.05, and 0.05, respectively. The primary shielding mechanism of our metal meshes is reflection, as indicated by the high reflection coefficients, where about 95% of incident energy is reflected by the mesh. Absorption plays a secondary role, where it is responsible for about 5% of the energy dissipation. However, absorption plays a crucial role in dissipating the remaining energy that is not reflected. The high absorption shielding efficiency indicates that the metal mesh is very effective at absorbing the small remaining amount of energy that is not reflected.

The results of *T*_550_, *R*_s_, σ_DC_/σ_OP_, haze, and EMI SE of the five samples are provided in Table 2.

**Table 2:** Transparent Electrode and EMI Shielding Performance of the Five Samples

sample	*T*_550_ (%)	*R*_s_ (Ω/sq)	σ_DC_/σ_OP_	haze (%)	SE (dB)
1	87.5	0.48	5690	36.3	42.5
2	91.3	0.54	7500	10.9	42.0
3	93.7	1.40	4070	7.0	37.4
4	90.2	0.66	5400	18.0	39.7
5	92.9	1.23	4090	7.2	38.3

**Figure 7:**
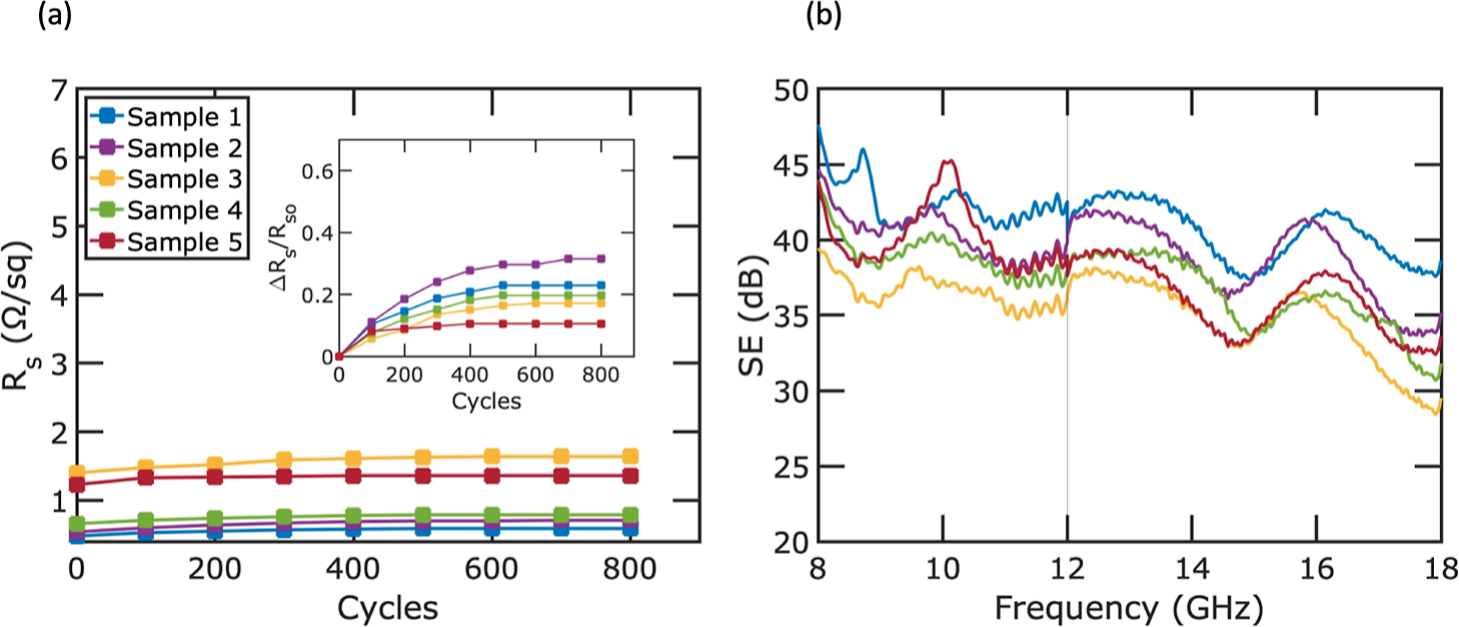
(a) Sheet resistance change of flexible metal mesh samples under bending test (inset: relative sheet resistance change vs bending cycles) and (b) EMI SE measurement for five samples after bending test.

In order to evaluate the mechanical durability of the embedded metal mesh structures for flexible optoelectronics, we conducted bending tests to assess the performance and resilience of the fabricated metal meshes. Figure 7a displays the results of the bending tests. Sheet resistance was measured after every 100 cycles of bending and reported as the absolute sheet resistance. The bending radius was 4 mm, and the samples were bent under tension. Each cycle represents one cycle of bending under tension and releasing this tension. In the initial phases of testing, all samples exhibited an increase in the sheet resistance. However, as the number of cycles increased, the sheet resistance values eventually reached a stable level for each sample. After undergoing 800 bending cycles, samples 1–5 exhibited an increase of 0.11, 0.17, 0.24, 0.13, and 0.13 Ω/sq in sheet resistance, respectively. Despite these increases in sheet resistance, the impact on the EMI SE was not significantly pronounced (Figure 7b). After the bending tests, the average SE for samples 1 through 5 changed from 42.5 to 41.4, from 42.0 to 39.5, from 37.4 to 35.6, from 39.7 to 37.6, and from 38.3 to 38.1 dB, respectively. These results provide insights into the mechanical resilience of our fabricated metal mesh structures in applications that require flexibility. To assess the adhesion strength between silver and the PET substrate, we conducted a pull-off adhesion test. This involved applying a thin, uniform layer of silver to PET and then measuring the force required to detach it. The details of this test are provided in Figure S5. When we applied an average stress of 2.7 MPa, the PET and the adhesive used in the test separated, instead of the silver and the PET. At a stress of 2.7 MPa, the tests resulted in a failure in the PET-glue bond as opposed to the silver and PET. This indicates that the silver bond with the PET is very strong with an adhesion strength of at least 2.7 MPa. These results demonstrate the silver ink’s robust adhesion to PET, confirming its reliability for use in flexible optoelectronic devices.

**Figure 8:**
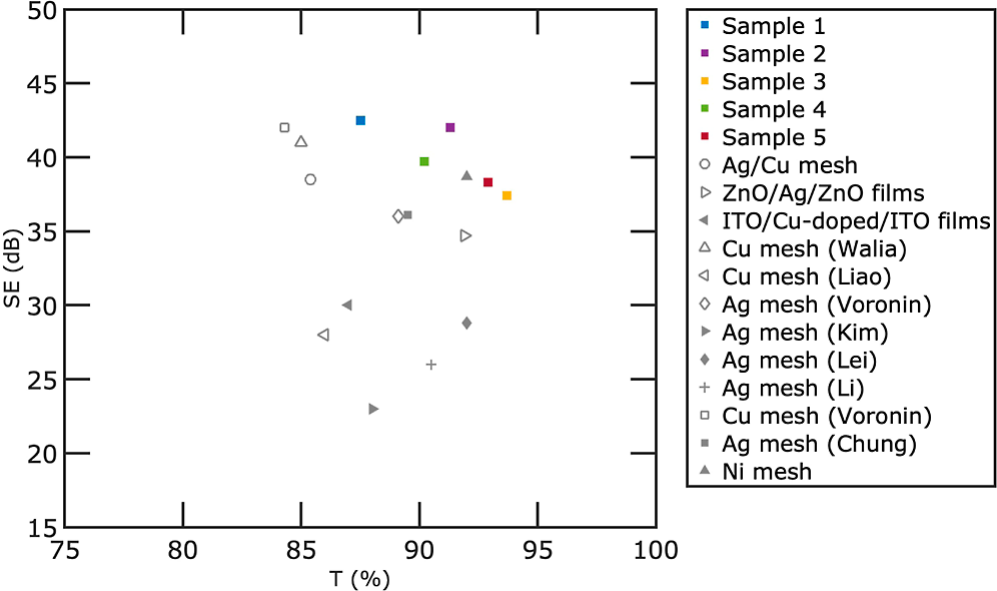
Comparison of the EMI SE (dB) and transmission (at 550 nm) of our flexible Ag meshes with those of other flexible metal meshes for EMI shielding in the literature,^57^ ZnO/Ag/ZnO films,^28^ ITO/Cu-doped/ITO films,^36^ Cu mesh (Walia),^56^ Cu mesh (Liao),^82^ Ag mesh (Voronin),^58^ Ag mesh (Kim),^65^ Ag mesh (Lei),^46^ Ag mesh (Li),^55^ Cu mesh (Voronin),^59^ Ag mesh (Chung),^51^ and Ni mesh.^29^

Figure 8 provides a comparative analysis of electromagnetic EMI SE and transmission of our flexible Ag meshes with those of other flexible metal meshes in the literature. Please note that in contrast to Figure 5, which only focuses on transparent electrodes fabricated by crack lithography, in this figure, we include metal meshes fabricated for flexible transparent EMI shielding purposes from various approaches. The studies we refer to include Ag/Cu mesh,^57^ ZnO/Ag/ZnO films,^28^ ITO/Cu-doped/ITO films,^36^ Cu mesh (Walia),^56^ Cu mesh (Liao),^82^ Ag mesh (Voronin),^58^ Ag mesh (Kim),^65^ Ag mesh (Lei),^46^ Ag mesh (Li),^55^ and Cu mesh (Voronin),^59^ Ag mesh (Chung),^51^ and Ni mesh.^29^ It is important to highlight that different studies have explored a diverse range of frequencies and have reported SE values in various formats such as maximum, minimum, or average. To maintain consistency in our analysis, we have chosen to report the average SE values within the frequency range of 8–18 GHz. For a more comprehensive comparison and additional details, please refer to Table S2 in the Supporting Information.

Previous research and simulations underscored the importance of having a small width and a substantial thickness in metal mesh structures to enhance EMI shielding efficiency.^43^ Unfortunately, the predominant manufacturing process, crack lithography, often results in metal meshes with thin layers of sputtered Ag, leading to suboptimal SE. Additionally, 3D printing techniques like electric-field-driven microscale,^55^ inkjet, gravure, screen, and flexography usually produce metal lines wider than 20 μm, which are not ideal for high-performance applications. However, we successfully achieved reduced width dimensions by carefully controlling the curing temperature and adjusting the template film thickness, leading to line widths under 5 μm.

Manufacturing metal meshes with significant thickness presents its own set of difficulties. Electroplating and electrodeposition methods are potential techniques for augmenting the thickness of metal meshes. However, they have limitations, such as the tendency of metal to deposit isotropically, which results in an unintended increase in width. In contrast, we are able to create trench depths over 5 μm in depth in PET substrates. The superior performance of our samples can be attributed to a combination of several factors, such as small width, large thickness, and the utilization of Ag ink, known for its higher conductivity in comparison to that of other metals and composite materials.^83^ Our method is capable of fabricating metal meshes with a small width and large thickness. The large thickness of the meshes results in more silver filling of the trenches, resulting in a lower sheet resistance and higher SE, while keeping the same visible transparency.

## Conclusions

In conclusion, this experimental study introduced a novel and straightforward approach for creating self-forming cracks as flexible, transparent EMI shielding, and transparent electrodes. The proposed method involved the transfer of self-forming cracks to a PET substrate using reactive-ion etching, followed by the filling of these cracks with silver ink. The study investigated the influence of curing temperature and template film thickness on the characteristics of the cracks, such as width, pitch, formation, and uniformity. The flexible embedded silver meshes demonstrated transparencies of 91.3 and 93.7% and corresponding *R*_s_ values of 0.54 and 1.40 Ω/sq, respectively. These correspond to σ_DC_/σ_OP_ values of 7500 and 4070, respectively. The samples demonstrate 42 dB SE with 91.3% transmission and 37.4 dB SE with 93.7% transmission. Notably, the results of this study surpass those of previous crack lithography works in the literature in terms of transparent electrode performance and all flexible transparent EMI shielding performance, regardless of fabrication approach. Furthermore, the elimination of the sputtering process in this method opens up promising prospects for a wide range of applications, including transparent electrodes, EMI shielding, solar cells, and organic light-emitting diodes.

## Experimental Section

### Ink Information

Commercially available EI-1201, a Ag metal-complex-based conductive ink from Electroninks Inc., was used in this study. The ink is was previously developed by one of the authors.^84^ In the ink, the ammonia ligand compounds act as a stabilizer. When the ink is cured in an oven, the liable ammonia ligand compounds evaporate, and the Ag compound is reduced to form Ag.

### Fabrication of PET-Embedded Ag Mesh

The fabrication of PET-embedded Ag meshes is described in Figure 1. PET sheets (MELINEX ST505) with a thickness of 125 μm were purchased from Tekra Inc. These sheets were cut into 30 mm × 30 mm samples using a scissor.

They were subjected to ultrasonic cleaning in acetone, methanol, and isopropyl alcohol (IPA) for 10 min each, followed by drying with nitrogen gas. Carboset CA-600 acrylic emulsion polymer was purchased from Lubrizol for use as the crack template. CA-600 is a water-based, 100% acrylic emulsion with a 41% volume solid content. Due to the settling of particles in the emulsion, we used ultrasonication to homogenize the solution. The acrylic emulsion was applied via spin coating on PET, with spin speeds meticulously set at 1000, 1500, and 2000 rpm. Cracks form during the curing process at temperatures of 60, 80, and 100 °C for various samples. Self-forming cracks was transferred to PET through RIE with a gas flow of 50 sccm CF_4_ and 20 sccm SF_6_, a pressure of 30 mT, and a power of 250 W. The samples were etched for the maximum allowable time to achieve the deepest trenches for each sample. During the etching process, defects begin to develop on the surface of the crack template film. As the etching time increases, these defects gradually penetrate the template film. Once these defects fully penetrate the template film, further etching begins to affect the PET substrate, creating small holes in PET. These unintended holes can trap silver ink during curing, thereby reducing transmission. Thus, there is a limitation on etching time, which depends on the thickness of the template film. After etching, the samples were subjected to ultrasonication in acetone to completely remove the template film and any other debris generated during the RIE process. Particle-free silver ink (EI-1201 from Electroninks) was drop casted on the samples and then ramp-cured starting at 70 °C with a step increase of 10 °C every 15 min. The final curing temperature was set at 110 °C. Prior to the hard-curing process, Ag was wiped off the sample using a cleanroom wiper following soft curing of Ag. Subsequently, a final curing step lasting 30 min was carried out to ensure the complete curing of Ag. The samples were then spray-washed with acetone, methanol, and IPA to remove any Ag particulates.

### Characterization

A probe station with a semiconductor device analyzer (B1500A Semiconductor Device Analyzer, Keysight Technologies) was used to measure sheet resistance via the van der Pauw method. To get high-resolution images of the PET-embedded Ag meshes, SEM (Zeiss SIGMA VP) was employed. The total transmittance was measured over the wavelength range of 400–800 nm using a UV–vis–(near-infrared) NIR spectrometer with a 100 mm diameter integrating sphere (PerkinElmer Lambda 750). The transmission values reported in our study were calculated by excluding the effect of bare PET transmission. To achieve this, we divided the measured transmission values by the bare PET transmission.

The EMI SE was determined using the coaxial transmission line method with the aid of an HP 7822D Vector Network Analyzer (VNA) for signal generation and detection. For the test, the samples 3 cm × 3 cm in size were positioned between two waveguide flanges, with the appropriate flange chosen based on the targeted frequency range. Specifically, we used the Pasternack WR-90 UG-135/U Square cover flange for the 8–12 GHz (X band) and the Pasternack WR-62 UG-1665/U Square cover flange for the 12–18 GHz range (Ku band). To ensure stability during measurement, the waveguide flanges were firmly attached using screws and nuts.

## Supporting Information Available

Comparison tables of the performance of our metal meshes with that of other metal meshes in the literature for EMI shielding and transparent electrodes, width distribution plots, image processing for centroid detection, depth measurement by optical profilometry, reflection and absorption SE contributions, and pull-off adhesion test (PDF)

## References

[ref1] Li M.; Sinha S.; Hannani S.; Walker S. B.; LeMieux M.; Leu P. W. Ink-Coated Silver Films on Pet for Flexible, High Performance Electromagnetic Interference Shielding and Joule Heating. ACS Appl. Electron. Mater. 2023, 5, 173–180. 10.1021/acsaelm.2c01183.

[ref2] Bhattacharjee Y.; Bose S. Core–Shell Nanomaterials for Microwave Absorption and Electromagnetic Interference Shielding: a Review. ACS Appl. Nano Mater. 2021, 4, 949–972. 10.1021/acsanm.1c00278.

[ref3] Li M.; Zarei M.; Galante A. J.; Pilsbury B.; Walker S. B.; LeMieux M.; Leu P. W. Stretchable and Wash Durable Reactive Silver Ink Coatings for Electromagnetic Interference Shielding, Joule Heating, and Strain Sensing Textiles. Prog. Org. Coat. 2023, 179, 107506 10.1016/j.porgcoat.2023.107506.

[ref4] Wang X.-Y.; Liao S.-Y.; Wan Y.-J.; Zhu P.-L.; Hu Y.-G.; Zhao T.; Sun R.; Wong C.-P. Electromagnetic interference shielding materials: recent progress, structure design, and future perspective. J. Mater. Chem. C 2022, 10, 44–72. 10.1039/D1TC04702G.

[ref5] Galante A. J.; Pilsbury B. C.; Li M.; LeMieux M.; Liu Q.; Leu P. W. Achieving Highly Conductive, Stretchable, and Washable Fabric from Reactive Silver Ink and Increased Interfacial Adhesion. ACS Appl. Polym. Mater. 2022, 4, 5253–5260. 10.1021/acsapm.2c00768.

[ref6] Zhan Y.; Santillo C.; Meng Y.; Lavorgna M. Recent advances and perspectives on silver-based polymer composites for electromagnetic interference shielding. J. Mater. Chem. C 2023, 11, 859–892. 10.1039/D2TC03821H.

[ref7] Liang C.; Gu Z.; Zhang Y.; Ma Z.; Qiu H.; Gu J. Structural Design Strategies of Polymer Matrix Composites for Electromagnetic Interference Shielding: A Review. Nano-Micro Lett. 2021, 13, 181 10.1007/s40820-021-00707-2. PMC8374026 34406529

[ref8] Li Z.; Li H.; Zhu X.; Peng Z.; Zhang G.; Yang J.; Wang F.; Zhang Y.-F.; Sun L.; Wang R.; Zhang J.; Yang Z.; Yi H.; Lan H. Directly Printed Embedded Metal Mesh for Flexible Transparent Electrode via Liquid Substrate Electric-Field-Driven Jet. Advanced Science 2022, 9, 2105331 10.1002/advs.202105331. 35233960 PMC9108624

[ref9] Li H.; Li Z.; Li N.; Zhu X.; Zhang Y.-F.; Sun L.; Wang R.; Zhang J.; Yang Z.; Yi H.; Xu X.; Lan H. 3d Printed High Performance Silver Mesh for Transparent Glass Heaters Through Liquid Sacrificial Substrate Electric-Field-Driven Jet. Small 2022, 18, 2107811 10.1002/smll.202107811. 35224846

[ref10] He Q.-M.; Tao J.-R.; Yang D.; Yang Y.; Wang M. Surface wrinkles enhancing electromagnetic interference shielding of copper coated polydimethylsiloxane: A simulation and experimental study. Chem. Eng. J. 2023, 454, 140162 10.1016/j.cej.2022.140162.

[ref11] Wang H.; Zheng D.; Zhang Y.; Han L.; Cao Z.; Lu Z.; Tan J. High-Performance Transparent Ultrabroadband Electromagnetic Radiation Shielding from Microwave toward Terahertz. ACS Appl. Mater. Interfaces 2023, 15, 49487–49499. 10.1021/acsami.3c10474. 37816124

[ref12] Lu X.; Zhang Y.; Zheng Z. Metal-Based Flexible Transparent Electrodes: Challenges and Recent Advances. Adv. Electron. Mater. 2021, 7, 2001121 10.1002/aelm.202001121.

[ref13] Nguyen V. H.; Papanastasiou D. T.; Resende J.; Bardet L.; Sannicolo T.; Jiménez C.; Muñoz-Rojas D.; Nguyen N. D.; Bellet D. Advances in Flexible Metallic Transparent Electrodes. Small 2022, 18, 2106006 10.1002/smll.202106006. 35195360

[ref14] Lee H. B.; Jin W.-Y.; Ovhal M. M.; Kumar N.; Kang J.-W. Flexible transparent conducting electrodes based on metal meshes for organic optoelectronic device applications: a review. J. Mater. Chem. C 2019, 7, 1087–1110. 10.1039/C8TC04423F.

[ref15] Li Z.; Li H.; Zhu X.; Peng Z.; Zhang G.; Yang J.; Wang F.; Zhang Y.; Sun L.; Wang R.; Zhang J.; Yang Z.; Yi H.; Lan H. Directly Printed Embedded Metal Mesh for Flexible Transparent Electrode via Liquid Substrate Electric-Field-Driven Jet. Advanced Science 2022, 9, 2105331 10.1002/advs.202105331. 35233960 PMC9108624

[ref16] Zhu X.; Liu M.; Qi X.; Li H.; Zhang Y.; Li Z.; Peng Z.; Yang J.; Qian L.; Xu Q.; Gou N.; He J.; Li D.; Lan H. Templateless, Plating-Free Fabrication of Flexible Transparent Electrodes with Embedded Silver Mesh by Electric-Field-Driven Microscale 3D Printing and Hybrid Hot Embossing. Adv. Mater. 2021, 33, 2007772 10.1002/adma.202007772. 33829552

[ref17] Xing Y.; Wan Y.; Wu Z.; Wang J.; Jiao S.; Liu L. Multilayer Ultrathin MXene@AgNW@MoS _2_ Composite Film for High-Efficiency Electromagnetic Shielding. ACS Appl. Mater. Interfaces 2023, 15, 5787–5797. 10.1021/acsami.2c18759. 36669167

[ref18] Chen Q.; Huang L.; Wang X.; Yuan Y. Transparent and Flexible Composite Films with Excellent Electromagnetic Interference Shielding and Thermal Insulating Performance. ACS Appl. Mater. Interfaces 2023, 15, 24901–24912. 10.1021/acsami.3c03140. 37171214

[ref19] Zhu M.; Yan X.; Li X.; Dai L.; Guo J.; Lei Y.; Xu Y.; Xu H. Flexible, Transparent, and Hazy Composite Cellulosic Film with Interconnected Silver Nanowire Networks for EMI Shielding and Joule Heating. ACS Appl. Mater. Interfaces 2022, 14, 45697–45706. 10.1021/acsami.2c13035. 36178711

[ref20] Lu Y.; Zhao X.; Lin Y.; Li P.; Tao Y.; Wang Z.; Ma J.; Xu H.; Liu Y. Lightweight MXene/carbon composite foam with hollow skeleton for air-stable, high-temperature-resistant and compressible electromagnetic interference shielding. Carbon 2023, 206, 375–382. 10.1016/j.carbon.2023.02.061.

[ref21] Gui H.; Zhao X.; Zuo S.; Liu W.; Wang C.; Xu P.; Ding Y.; Yao C. Carbonized Syndiotactic Polystyrene/Carbon Nanotube/MXene Hybrid Aerogels with Egg-Box Structure: A Platform for Electromagnetic Interference Shielding and Solar Thermal Energy Management. ACS Appl. Mater. Interfaces 2023, 15, 39740–39751. 10.1021/acsami.3c08176. 37556599

[ref22] Xue T.; Yang Y.; Yu D.; Wali Q.; Wang Z.; Cao X.; Fan W.; Liu T. 3D Printed Integrated Gradient-Conductive MXene/CNT/Polyimide Aerogel Frames for Electromagnetic Interference Shielding with Ultra-Low Reflection. Nano-Micro Lett. 2023, 15, 45 10.1007/s40820-023-01017-5. PMC9908813 36752927

[ref23] Guo Z.; Ren P.; Yang F.; Wu T.; Zhang L.; Chen Z.; Huang S.; Ren F. MOF-Derived Co/C and MXene *co* -Decorated Cellulose-Derived Hybrid Carbon Aerogel with a Multi-Interface Architecture toward Absorption-Dominated Ultra-Efficient Electromagnetic Interference Shielding. ACS Appl. Mater. Interfaces 2023, 15, 7308–7318. 10.1021/acsami.2c22447. 36693013

[ref24] Cheng M.; Ying M.; Zhao R.; Ji L.; Li H.; Liu X.; Zhang J.; Li Y.; Dong X.; Zhang X. Transparent and Flexible Electromagnetic Interference Shielding Materials by Constructing Sandwich AgNW@MXene/Wood Composites. ACS Nano 2022, 16, 16996–17007. 10.1021/acsnano.2c07111. 36134706

[ref25] Bian X.; Yang Z.; Zhang T.; Yu J.; Xu G.; Chen A.; He Q.; Pan J. Multifunctional Flexible AgNW/MXene/PDMS Composite Films for Efficient Electromagnetic Interference Shielding and Strain Sensing. ACS Appl. Mater. Interfaces 2023, 15, 41906–41915. 10.1021/acsami.3c08093. 37610108

[ref26] Liang L.; Li Q.; Yan X.; Feng Y.; Wang Y.; Zhang H.-B.; Zhou X.; Liu C.; Shen C.; Xie X. Multifunctional Magnetic Ti _3_ C _2_ T _x_ MXene/Graphene Aerogel with Superior Electromagnetic Wave Absorption Performance. ACS Nano 2021, 15, 6622–6632. 10.1021/acsnano.0c09982. 33780231

[ref27] Wang H.; Ji C.; Zhang C.; Zhang Y.; Zhang Z.; Lu Z.; Tan J.; Guo L. J. Highly Transparent and Broadband Electromagnetic Interference Shielding Based on Ultrathin Doped Ag and Conducting Oxides Hybrid Film Structures. ACS Appl. Mater. Interfaces 2019, 11, 11782–11791. 10.1021/acsami.9b00716. 30817123

[ref28] Yuan C.; Huang J.; Dong Y.; Huang X.; Lu Y.; Li J.; Tian T.; Liu W.; Song W. Record-High Transparent Electromagnetic Interference Shielding Achieved by Simultaneous Microwave Fabry–Pérot Interference and Optical Antireflection. ACS Appl. Mater. Interfaces 2020, 12, 26659–26669. 10.1021/acsami.0c05334. 32422036

[ref29] Jiang Z.-Y.; Huang W.; Chen L.-S.; Liu Y.-H. Ultrathin, Lightweight, and Freestanding Metallic Mesh for Transparent Electromagnetic Interference Shielding. Opt. Express 2019, 27, 24194–24206. 10.1364/oe.27.024194. 31510313

[ref30] He J.; Li A.; Wang W.; Cui C.; Jiang S.; Chen M.; Qin W.; Tang H.; Guo R. Multifunctional Wearable Device Based on an Antibacterial and Hydrophobic Silver Nanoparticles/Ti _3_ C _2_ T _x_ MXene/Thermoplastic Polyurethane Fibrous Membrane for Electromagnetic Shielding and Strain Sensing. Ind. Eng. Chem. Res. 2023, 62, 9221–9232. 10.1021/acs.iecr.3c00214.

[ref31] Mai T.; Guo W.-Y.; Wang P.-L.; Chen L.; Qi M.-Y.; Liu Q.; Ding Y.; Ma M.-G. Bilayer metal-organic frameworks/MXene/nanocellulose paper with electromagnetic double loss for absorption-dominated electromagnetic interference shielding. Chem. Eng. J. 2023, 464, 142517 10.1016/j.cej.2023.142517.

[ref32] Tran V. V.; Nguyen D. D.; Nguyen A. T.; Hofmann M.; Hsieh Y.-P.; Kan H.-C.; Hsu C.-C. Electromagnetic Interference Shielding by Transparent Graphene/Nickel Mesh Films. ACS Appl. Nano Mater. 2020, 3, 7474–7481. 10.1021/acsanm.0c01076.

[ref33] Fu H.; Chen L.; Liu D.; Zhang Y.; Cao Y.; Wu C.; Yong Z.; Yu Y.; Li Q. Multifunctional NiCo@RGO/SWNTs foam with oriented pore structure for excellent electromagnetic interference shielding. Chem. Eng. J. 2023, 454, 140324 10.1016/j.cej.2022.140324.

[ref34] Wanasinghe D.; Aslani F. A review on recent advancement of electromagnetic interference shielding novel metallic materials and processes. Composites, Part B 2019, 176, 107207 10.1016/j.compositesb.2019.107207.

[ref35] Li M.; McCourt M. J.; Galante A. J.; Leu P. W. Bayesian Optimization of Nanophotonic Electromagnetic Shielding with Very High Visible Transparency. Opt. Express 2022, 30, 33182–33194. 10.1364/OE.468843. 36242364

[ref36] Wang H.; Ji C.; Zhang C.; Zhang Y.; Zhang Z.; Lu Z.; Tan J.; Guo L. J. Highly Transparent and Broadband Electromagnetic Interference Shielding Based on Ultrathin Doped Ag and Conducting Oxides Hybrid Film Structures. ACS Appl. Mater. Interfaces 2019, 11, 11782–11791. 10.1021/acsami.9b00716. 30817123

[ref37] Kim J.; Li M.; Li Y.; Gomez A.; Hinder O.; Leu P. W. Multi-BOWS: Multi-Fidelity Multi-Objective Bayesian Optimization with Warm Starts for Nanophotonic Structure Design. Digital Discovery 2024, 10.1039/d3dd00177f.

[ref38] Jiang Z.; Zhao S.; Chen L.; Liu Y.-h. Freestanding “core-shell” AgNWs/metallic hybrid mesh electrodes for a highly efficient transparent electromagnetic interference shielding film. Opt. Express 2021, 29, 18760 10.1364/OE.423369. 34154125

[ref39] Gu J.; Hu S.; Ji H.; Feng H.; Zhao W.; Wei J.; Li M. Multi-layer silver nanowire/polyethylene terephthalate mesh structure for highly efficient transparent electromagnetic interference shielding. Nanotechnology 2020, 31, 185303 10.1088/1361-6528/ab6d9d. 31958779

[ref40] Zhu X.; Guo A.; Yan Z.; Qin F.; Xu J.; Ji Y.; Kan C. PET/Ag NW/PMMA transparent electromagnetic interference shielding films with high stability and flexibility. Nanoscale 2021, 13, 8067–8076. 10.1039/D1NR00977J. 33881446

[ref41] Yang H.; Bai S.; Guo X.; Wang H. Robust and smooth UV-curable layer overcoated AgNW flexible transparent conductor for EMI shielding and film heater. Appl. Surf. Sci. 2019, 483, 888–894. 10.1016/j.apsusc.2019.04.034.

[ref42] Zarei M.; Loy J. C.; Li M.; Zhou Z.; Sinha S.; LeMieux M.; Walker S. B.; Rand B. P.; Leu P. W. Substrate-embedded metal meshes for ITO-free organic light emitting diodes. Opt. Express 2023, 31, 34697 10.1364/OE.499932. 37859220

[ref43] Li M.; Zarei M.; Mohammadi K.; Walker S. B.; LeMieux M.; Leu P. W. Silver Meshes for Record-Performance Transparent Electromagnetic Interference Shielding. ACS Appl. Mater. Interfaces 2023, 15, 30591–30599. 10.1021/acsami.3c02088. 37314726 PMC10316324

[ref44] Wang H.; Lu Z.; Liu Y.; Tan J.; Ma L.; Lin S. Double-Layer Interlaced Nested Multi-Ring Array Metallic Mesh for High-Performance Transparent Electromagnetic Interference Shielding. Opt. Lett. 2017, 42, 1620–1623. 10.1364/OL.42.001620. 28409813

[ref45] Liang Z.; Zhao Z.; Pu M.; Luo J.; Xie X.; Wang Y.; Guo Y.; Ma X.; Luo X. Metallic nanomesh for high-performance transparent electromagnetic shielding. Opt. Mater. Express 2020, 10, 796 10.1364/OME.386830.

[ref46] Lei Q.; Luo Z.; Zheng X.; Lu N.; Zhang Y.; Huang J.; Yang L.; Gao S.; Liang Y.; He S. Broadband Transparent and Flexible Silver Mesh for Efficient Electromagnetic Interference Shielding and High-Quality Free-Space Optical Communication. Opt. Mater. Express 2023, 13, 469–483. 10.1364/ome.478830.

[ref47] Liang Y.; Huang X.; Wen K.; Wu Z.; Yao L.; Pan J.; Liu W.; Liu P. Metal Mesh-Based Infrared Transparent EMI Shielding Window with Balanced Shielding Properties over a Wide Frequency Spectrum. Appl. Sci. 2023, 13, 4846 10.3390/app13084846.

[ref48] Han Y.; Zhong H.; Liu N.; Liu Y.; Lin J.; Jin P. In Situ Surface Oxidized Copper Mesh Electrodes for High-Performance Transparent Electrical Heating and Electromagnetic Interference Shielding. Adv. Electron. Mater. 2018, 4, 1800156 10.1002/aelm.201800156.

[ref49] Ma L.; Lu Z.; Tan J.; Liu J.; Ding X.; Black N.; Li T.; Gallop J.; Hao L. Transparent Conducting Graphene Hybrid Films to Improve Electromagnetic Interference (EMI) Shielding Performance of Graphene. ACS Appl. Mater. Interfaces 2017, 9, 34221–34229. 10.1021/acsami.7b09372. 28892351

[ref50] Zhang Y.; Dong H.; Li Q.; Mou N.; Chen L.; Zhang L. Double-layer metal mesh etched by femtosecond laser for high-performance electromagnetic interference shielding window. RSC Adv. 2019, 9, 22282–22287. 10.1039/C9RA03519B. 35519472 PMC9066645

[ref51] Chung S.-i.; Kim P. K.; Ha T.-g. High-performance transparent electromagnetic interference shielding film based on metal meshes. J. Micromech. Microeng. 2023, 33, 035002 10.1088/1361-6439/acb65e.

[ref52] Chung S.-i.; Kang T.-W.; Kim P. K.; Ha T.-g.; Hong Y.-P. Highly Transparent Ka-/W-Band Electromagnetic Shielding Films Based on Double-Layered Metal Meshes. ACS Appl. Mater. Interfaces 2023, 15, 56612–56622. 10.1021/acsami.3c14788. 37988133

[ref53] Shen S.; Chen S.-Y.; Zhang D.-Y.; Liu Y.-H. High-performance composite Ag-Ni mesh based flexible transparent conductive film as multifunctional devices. Opt. Express 2018, 26, 27545 10.1364/OE.26.027545. 30469819

[ref54] Li H.; Zhang Y.; Tai Y.; Zhu X.; Qi X.; Zhou L.; Li Z.; Lan H. Flexible Transparent Electromagnetic Interference Shielding Films with Silver Mesh Fabricated Using Electric-Field-Driven Microscale 3d Printing. Opt Laser. Technol. 2022, 148, 107717 10.1016/j.optlastec.2021.107717.

[ref55] Li H.; Zhang Y.; Tai Y.; Zhu X.; Qi X.; Zhou L.; Li Z.; Lan H. Flexible transparent electromagnetic interference shielding films with silver mesh fabricated using electric-field-driven microscale 3D printing. Opt Laser. Technol. 2022, 148, 107717 10.1016/j.optlastec.2021.107717.

[ref56] Walia S.; Singh A. K.; Rao V. S. G.; Bose S.; Kulkarni G. U. Metal Mesh-Based Transparent Electrodes as High-Performance EMI Shields. Bull. Mater. Sci. 2020, 43, 187 10.1007/s12034-020-02159-7.

[ref57] Voronin A. S.; Fadeev Y. V.; Govorun I. V.; Podshivalov I. V.; Simunin M. M.; Tambasov I. A.; Karpova D. V.; Smolyarova T. E.; Lukyanenko A. V.; Karacharov A. A.; Nemtsev I. V.; Khartov S. V. Cu–Ag and Ni–Ag Meshes Based on Cracked Template as Efficient Transparent Electromagnetic Shielding Coating with Excellent Mechanical Performance. J. Mater. Sci. 2021, 56, 14741–14762. 10.1007/s10853-021-06206-4.

[ref58] Voronin A.; Fadeev Y.; Ivanchenko F.; Dobrosmyslov S.; Makeev M.; Mikhalev P.; Osipkov A.; Damaratsky I.; Ryzhenko D.; Yurkov G.; et al. Original concept of cracked template with controlled peeling of the cells perimeter for high performance transparent EMI shielding films. Surf. Interfaces 2023, 38, 102793 10.1016/j.surfin.2023.102793.

[ref59] Voronin A. S.; Fadeev Y. V.; Makeev M. O.; Mikhalev P. A.; Osipkov A. S.; Provatorov A. S.; Ryzhenko D. S.; Yurkov G. Y.; Simunin M. M.; Karpova D. V.; et al. Low Cost Embedded Copper Mesh Based on Cracked Template for Highly Durability Transparent EMI Shielding Films. Materials 2022, 15, 1449 10.3390/ma15041449. 35207987 PMC8879047

[ref60] Han Y.; Liu Y.; Han L.; Lin J.; Jin P. High-performance hierarchical graphene/metal-mesh film for optically transparent electromagnetic interference shielding. Carbon 2017, 115, 34–42. 10.1016/j.carbon.2016.12.092.

[ref61] Han Y.; Lin J.; Liu Y.; Fu H.; Ma Y.; Jin P.; Tan J. Crackle template based metallic mesh with highly homogeneous light transmission for high-performance transparent EMI shielding. Sci. Rep. 2016, 6, 25601 10.1038/srep25601. 27151578 PMC4858675

[ref62] Yang Z.; Hao Q.; Zhang S.; Sun X.; Tian W.; Liu F. Multispectral transparent electromagnetic-wave-absorbing optical window technology based on a random grid. Opt. Express 2023, 31, 26355 10.1364/OE.497225. 37710498

[ref63] Jiang Z.; Zhao S.; Huang W.; Chen L.; Liu Y.-H. Embedded Flexible and Transparent Double-Layer Nickel-Mesh for High Shielding Efficiency. Opt. Express 2020, 28, 26531–26542. 10.1364/oe.401543. 32906925

[ref64] Lu Z.; Ma L.; Tan J.; Wang H.; Ding X. Graphene, microscale metallic mesh, and transparent dielectric hybrid structure for excellent transparent electromagnetic interference shielding and absorbing. 2D Materials 2017, 4, 025021 10.1088/2053-1583/aa57f8.

[ref65] Kim M.-H.; Joh H.; Hong S.-H.; Oh S. J. Coupled Ag nanocrystal-based transparent mesh electrodes for transparent and flexible electro-magnetic interference shielding films. Curr. Appl. Phys. 2019, 19, 8–13. 10.1016/j.cap.2018.10.016.

[ref66] Jung J.; Kim K. K.; Suh Y. D.; Hong S.; Yeo J.; Ko S. H. Recent progress in controlled nano/micro cracking as an alternative nano-patterning method for functional applications. Nanoscale Horiz. 2020, 5, 1036–1049. 10.1039/D0NH00241K. 32469038

[ref67] Liu P.; Huang B.; Peng L.; Liu L.; Gao Q.; Wang Y. A crack templated copper network film as a transparent conductive film and its application in organic light-emitting diode. Sci. Rep. 2022, 12, 20494 10.1038/s41598-022-24672-x. 36443395 PMC9705311

[ref68] Cui M.; Zhang X.; Rong Q.; Nian L.; Shui L.; Zhou G.; Li N. High conductivity and transparency metal network fabricated by acrylic colloidal self-cracking template for flexible thermochromic device. Org. Electron. 2020, 83, 105763 10.1016/j.orgel.2020.105763.

[ref69] Han B.; Pei K.; Huang Y.; Zhang X.; Rong Q.; Lin Q.; Guo Y.; Sun T.; Guo C.; Carnahan D.; Giersig M.; Wang Y.; Gao J.; Ren Z.; Kempa K. Uniform Self-Forming Metallic Network as a High-Performance Transparent Conductive Electrode. Adv. Mater. 2014, 26, 873–877. 10.1002/adma.201302950. 24510662

[ref70] Muzzillo C. P.; Reese M. O.; Lee C.; Xiong G. Cracked Film Lithography with CuGaO _x_ Buffers for Bifacial CdTe Photovoltaics. Small 2023, 19, 2301939 10.1002/smll.202301939. 37010046

[ref71] Xian Z.; Han B.; Li S.; Yang C.; Wu S.; Lu X.; Gao X.; Zeng M.; Wang Q.; Bai P.; Naughton M. J.; Zhou G.; Liu J.-M.; Kempa K.; Gao J. A Practical ITO Replacement Strategy: Sputtering-Free Processing of a Metallic Nanonetwork. Adv. Mater. Technol. 2017, 2, 1700061 10.1002/admt.201700061.

[ref72] Muzzillo C. P.; Reese M. O.; Mansfield L. M. Fundamentals of Using Cracked Film Lithography to Pattern Transparent Conductive Metal Grids for Photovoltaics. Langmuir 2020, 36, 4630–4636. 10.1021/acs.langmuir.0c00276. 32275439

[ref73] Melnychenko A. M.; Kudrawiec R. Crack-Templated Wire-Like Semitransparent Electrodes with Unique Irregular Patterns. ACS Omega 2022, 7, 39181–39186. 10.1021/acsomega.2c05131. 36340126 PMC9631720

[ref74] Kang S.; Arepalli V. K.; Yang E.; Lee S.; Wi J.-S.; Yun J. H.; Song S.; Kim K.; Eo Y.-J.; Cho J.-S.; Gwak J.; Chung C.-H. High Performance and Flexible Electrodeposited Silver Mesh Transparent Conducting Electrodes Based on a Self-Cracking Template. Electron. Mater. Lett. 2022, 18, 440–446. 10.1007/s13391-022-00358-4.

[ref75] Kayes M. I.; Zarei M. M.; Feng F.; Leu P. W. Black silicon spacing effect on bactericidal efficacy against gram-positive bacteria. Nanotechnology 2023, 35, 025102 10.1088/1361-6528/acfe16. 37769640

[ref76] Rao K. D. M.; Gupta R.; Kulkarni G. U. Fabrication of Large Area, High-Performance, Transparent Conducting Electrodes Using a Spontaneously Formed Crackle Network as Template. Adv. Mater. Interfaces 2014, 1, 1400090 10.1002/admi.201400090.

[ref77] Goehring L.; Nakahara A.; Dutta T.; Kitsunezaki S.; Tarafdar S. Desiccation Cracks and Their Patterns: Formation and Modelling in Science and Nature; John Wiley & Sons, 2015.

[ref78] Hutchinson J.; Suo Z. Advances in Applied Mechanics; Elsevier, 1991; Vol. 29, pp 63–191.

[ref79] Gupta R.; Rao K. D. M.; Srivastava K.; Kumar A.; Kiruthika S.; Kulkarni G. U. Spray Coating of Crack Templates for the Fabrication of Transparent Conductors and Heaters on Flat and Curved Surfaces. ACS Appl. Mater. Interfaces 2014, 6, 13688–13696. 10.1021/am503154z. 25001064

[ref80] Rao K. D. M.; Hunger C.; Gupta R.; Kulkarni G. U.; Thelakkat M. A cracked polymer templated metal network as a transparent conducting electrode for ITO-free organic solar cells. Phys. Chem. Chem. Phys. 2014, 16, 15107–15110. 10.1039/C4CP02250E. 24958552

[ref81] Guo C. F.; Sun T.; Liu Q.; Suo Z.; Ren Z. Highly Stretchable and Transparent Nanomesh Electrodes Made by Grain Boundary Lithography. Nat. Commun. 2014, 5, 3121 10.1038/ncomms4121. 24469072

[ref82] Liao D.; Zheng Y.; Ma X.; Fu Y. Honeycomb-ring hybrid random mesh design with electromagnetic interference (EMI) shielding for low stray light. Opt. Express 2023, 31, 32200 10.1364/OE.500407. 37859028

[ref83] Rosker E. S.; Barako M. T.; Nguyen E.; DiMarzio D.; Kisslinger K.; Duan D.-W.; Sandhu R.; Goorsky M. S.; Tice J. Approaching the Practical Conductivity Limits of Aerosol Jet Printed Silver. ACS Appl. Mater. Interfaces 2020, 12, 29684 10.1021/acsami.0c06959. 32496037

[ref84] Walker S. B.; Lewis J. A. Reactive Silver Inks for Patterning High-Conductivity Features at Mild Temperatures. J. Am. Chem. Soc. 2012, 134, 1419–1421. 10.1021/ja209267c. 22220580

