# Supplementary material for: Flexible Embedded Metal Meshes by Sputter-Free Crack Lithography for Transparent Electrodes and Electromagnetic Interference Shielding
Source: ACS Appl Mater Interfaces. 2024 Jan 27;16(5):6382–93. doi: 10.1021/acsami.3c16405 (PMC10859897; doi:10.1021/acsami.3c16405)
Supplement: Supplementary file 1 — am3c16405_si_001.pdf [file am3c16405_si_001.pdf]

Supporting Information for Flexible Embedded Metal Meshes by Sputter-Free Crack Lithography for Transparent Electrodes and Electromagnetic Interference Shielding

Mehdi Zarei,[†] Mingxuan Li,[‡] Elizabeth E. Medvedeva,[¶] Sooraj Sharma,[§] Jungtaek Kim,[‖] Zefan Shao,[†] S. Brett Walker,[⊥] Melbs LeMieux,[⊥] Qihan Liu,[†] and Paul W. Leu*,[‖,†,‡]

†*Department of Mechanical Engineering, University of Pittsburgh, Pittsburgh, PA 15261, United States*

‡*Department of Chemical Engineering, University of Pittsburgh, Pittsburgh, PA 15261, United States*

¶*Department of Bioengineering, University of Pittsburgh, Pittsburgh, PA 15261, United States*

§*Department of Materials Science, University of Pittsburgh, Pittsburgh, PA 15261, United States*

‖*Department of Industrial Engineering, University of Pittsburgh, Pittsburgh, PA 15261, United States*

⊥*Electroninks Incorporated, Austin, TX 78744, United States*

E-mail: pleu@pitt.edu

Table S1: Comparison of the performance of our metal meshes with other crack lithography metal meshes in the literature as transparent electrodes.

**Reference**	**Metal**	**Template**	**Subs.**	$\boldsymbol{T}$ **(%)**	$\boldsymbol{R_s}$ **(Ω/*sq*)**	$\boldsymbol{\sigma_{\mathrm{DC}}/\sigma_{\mathrm{OP}}}$
Sample 1	Ag	CA-600	PET	87.5	0.48	5688
Sample 2	Ag	CA-600	PET	91.3	0.54	7497
Sample 3	Ag	CA-600	PET	93.7	1.40	4071
Sample 4	Ag	CA-600	PET	90.2	0.66	5400
Sample 5	Ag	CA-600	PET	92.9	1.23	4086
Liu *et. al*[1]	Cu	acrylic resin	PET	93	13.4	380
Cui *et. al*[2]	Ag	acrylic resin	PET	86.39	6.08	409
Tran *et. al*[3]	Graphene/Ni	TiO2	glass	86	173	14
Melnychenko *et. al*[4]	Ti/Ag	ZnO/PVP	glass	78	13.65	104
Han *et. al*[5]	Ag	TiO2	PET	88	10	286
Rao *et. al*[6]	Ag	acrylic resin	PET	82.5	0.55	3395
Xian *et. al*[7]	Ag	egg white	PET	84.8	1.13	2000
Gupta *et. al*[8]	Ag	acrylic resin	PET	84	0.9	2445
Rao *et. al*[9]	Ag	acrylic resin	glass	86	10	241
Guo *et. al*[10]	Au	In2O3	PDMS	82.5	20	93
Kang *et. al*[11]	Ag	acrylic resin	PET	88	1.01	2827
Muzzillo *et. al*[12]	Au	PMMA	CdTe	76	8.3	154
Voronin *et. al*[13]	Ag	egg white	CdTe	79.3	0.28	5475

Table S2: EMI shielding performance comparison between our metal meshes and those in the existing literature.

**Reference**	**Material**	**Subs.**	$\boldsymbol{T}$ **(%)**	**Freq. (GHz)**	$\mathbf{SE_{ave}}$ **(dB)**
Sample 1	Ag mesh	PET	87.5	8-18	42.5
Sample 2	Ag mesh	PET	91.3	8-18	42
Sample 3	Ag mesh	PET	93.7	8-18	37.4
Sample 4	Ag mesh	PET	90.2	8-18	39.7
Sample 5	Ag mesh	PET	92.9	8-18	38.3
Voronin *et. al*[14]	Ag/Cu mesh	PET	85.4	8-12	38.5
Yuan *et. al*[15]	ZnO/Ag/ZnO	PET	91.9	8-18	34.7
Wang *et. al*[16]	ITO/Cu-doped/ITO	PET	87	8-18	30
Walia *et. al*[17]	Cu mesh	PET	85	12-18	41
Liao *et. al*[18]	Cu mesh	PET	86	12-18	28
Voronin *et. al*[13]	Ag mesh	PET	89.1	0-7	36
Kim *et. al*[19]	Ag mesh	PET	88	1.5-10	23
Lei *et. al*[20]	Ag mesh	PET	92	8-12	28.8
Li *et. al*[21]	Ag mesh	PET	90.5	0-3	26
Voronin *et. al*[22]	Cu mesh	PET	84.3	0-9	42
Chung *et. al*[23]	Cu mesh	PET	89.5	8-18	36.1
Jiang *et. al*[24]	Cu mesh	PET	92	8.2-12.4	38.7
Li *et. al*[25]	Ag mesh	glass	90.3	8-18	48.3
Ma *et. al*[26]	Cu/Graphene	glass	91	12-18	25.5
Jiang *et. al*[24]	Ni mesh/ITO	glass	92	8-12	40
Wang *et. al*[27]	Multi-ring Al meshes	glass	90	12-18	27
Liang *et. al*[28]	Cr/Cu mesh	glass	85	8-18	45
Liang *et. al*[29]	Cr/Au mesh	sapphire	85	8-18	23
Tran *et. al*[3]	Graphene/ Ni	glass	83	0.75-3	19
Han *et. al*[30]	Cu mesh	glass	88.2	12-18	24
Shen *et. al*[31]	Ag/Ni mesh	glass	83	8-12	43
Han *et. al*[32]	Graphene/Cu mesh	glass	90	12-18	14
Jiang *et. al*[33]	AgNW/Ni mesh	glass	93	8-12	41.5
Lu *et. al*[34]	Graphene/Cu mesh	glass	90	12-18	37.5
Han *et. al*[35]	Ag mesh	glass	91	0-5	26
Yang *et. al*[36]	Cu/Ni/Ti	glass	81	8-18	38

Table S2 shows the comparison of EMI shielding performance of our fabricated samples with other metal meshes in literature. The table is structured into three sections: the first segment presents samples derived from our research on PET (polyethylene terephthalate)

substrates; the second section highlights metal meshes from other studies applied to PET substrates; and the third section centers on metal meshes employed with either glass or sapphire substrates. This table provides information regarding the material type, the substrate applied, light transmission rate at 550 nm wavelength, examined frequency range, and the average shielding efficiency (SE_{ave}). It is worth noting that the references studied in the literature have explored various frequency ranges and reported SE values in terms of maximum, minimum, or average. To maintain consistency throughout our paper, we have opted to specifically report the average SE within the frequency range of 8 - 18 GHz.

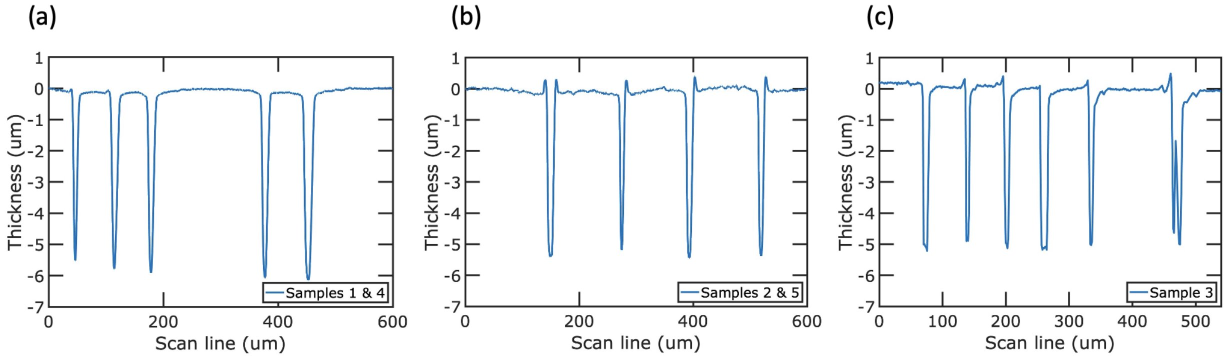

Figure S1: Depth of etched cracks in the five studied samples, determined by optical profilometry measurements, for different etch times of (a) 950, (b) 750, and (c) 650 seconds.

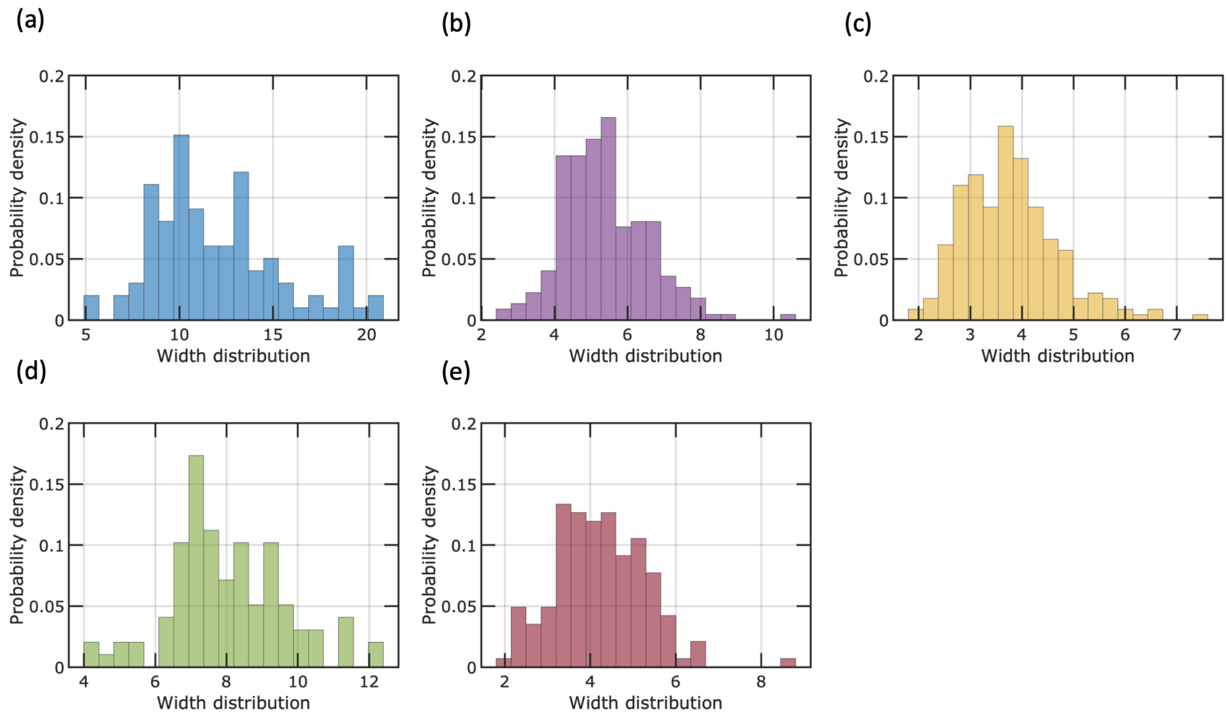

Figure S2: Probability density vs. width distribution for five fabricated samples , ranging from (a) to (e) for samples 1 through 5, respectively.

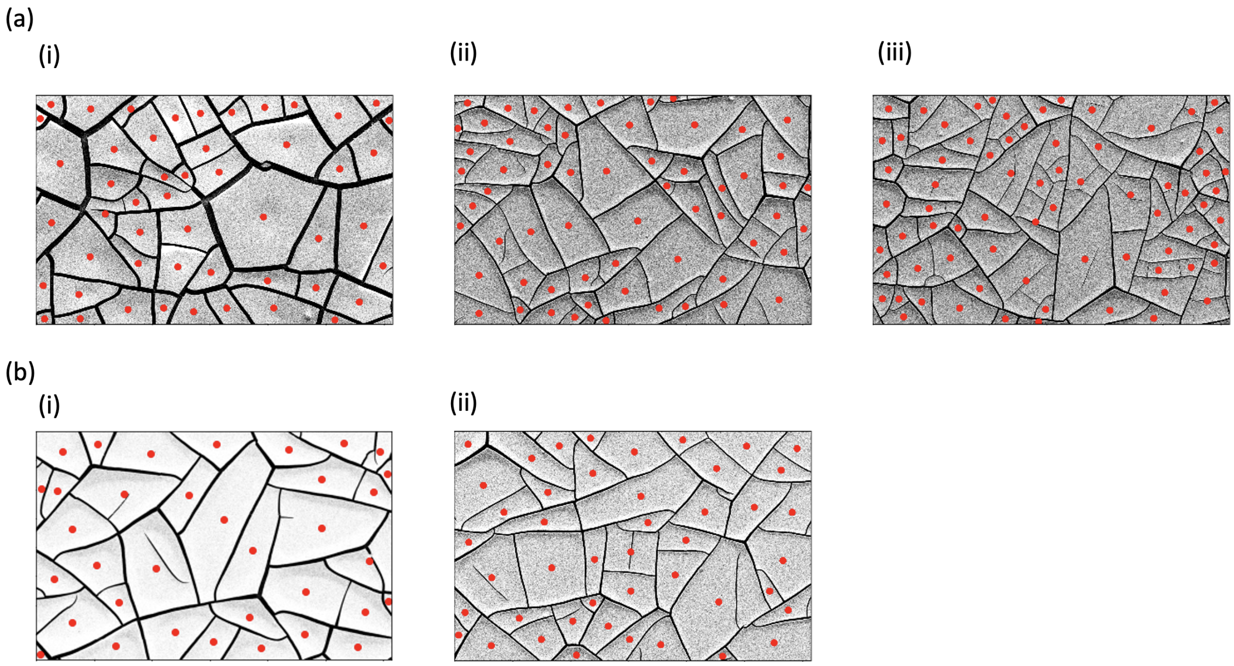

Figure S3: Centroid detection for each isolated cell analyzed through image processing for the five fabricated samples, ranging from (a) to (e) for samples 1 through 5, respectively.

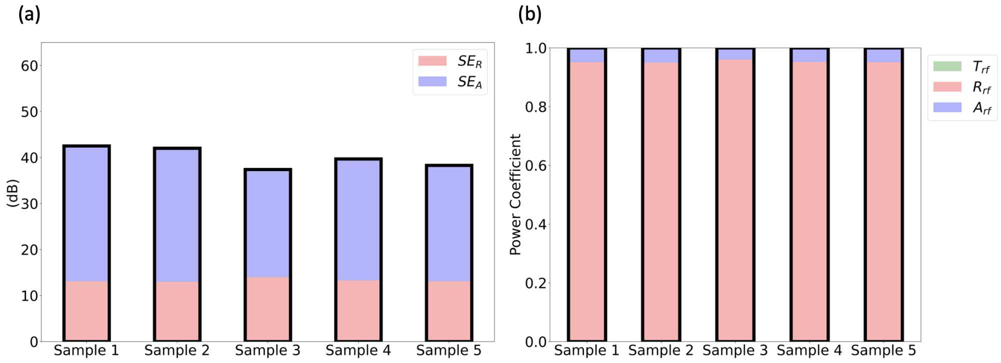

Figure S4: (a) SE contribution of SE_A and SE_R. (b) Power coefficient for five samples in the radio frequency.

Silver meshes provide for shielding through both reflection and absorption. Figure S4 shows the shielding efficiency contribution of the five samples, where the total shielding efficiency SE is a sum of the reflection shielding efficiency SE_R and absorption efficiency SE_A, $\mathrm{SE} = \mathrm{SE}_R + \mathrm{SE}_A$, where all are evaluated in the 8 - 18 GHz range. These shielding efficiencies are calculated using the following equations:

$$\mathrm{SE}_R = -10\log_{10}(1 - R_{rf}), \tag{S1}$$

where R_{rf} denotes the reflection coefficient and

$$\mathrm{SE}_A = -10\log_{10}\frac{T_{rf}}{(1 - R_{rf})} \tag{S2}$$

where T_{rf} denotes the transmission coefficient in the radio frequency. For the five samples, the reflection efficiencies SE_R are 13.1, 13.0, 14.0, 13.3, and 13.1 dB, respectively, while the absorption efficiencies SE_A are 29.4, 29.0, 23.4, 26.4, and 25.2 dB, respectively.

The coefficients of transmission (T_{rf}), reflection (R_{rf}), and absorption (A_{rf}) represent the proportions of an incident electromagnetic wave that are transmitted, reflected, and absorbed, respectively. These coefficients satisfy the following equation due to conservation

of energy:

$$T_{rf} + R_{rf} + A_{rf} = 1 \tag{S3}$$

The coefficients T_{rf} and R_{rf} are determined from the scattering matrix elements

$$T_{rf} = |S_{21}|^2 \tag{S4}$$

and

$$R_{rf} = |S_{11}|^2. \tag{S5}$$

The reflection coefficients for the five samples are 0.95, 0.95, 0.96, 0.95, 0.95, respectively, and the corresponding absorption coefficients are 0.05, 0.05, 0.04, 0.05, 0.05, respectively.

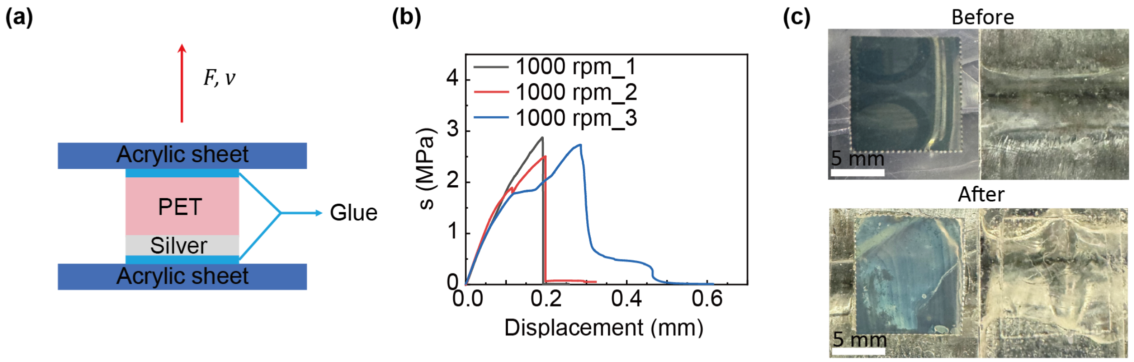

Figure S5: Characterizing of silver-PET adhesion test (a) schematic of the test set up (b) stress vs. displacement (c) optical images of the sample before and after the test.

Figure S5 presents the results of the adhesion test performed on silver-coated PET (polyethylene terephthalate) samples. The process began with a 3 cm by 3 cm PET sample, onto which silver ink was spin-coated at a speed of 1000 rpm to ensure a uniform layer of silver film. This sample underwent a ramp-cure process identical to that used for our metal meshes. The curing procedure started at a temperature of 70 °C, increasing incrementally by 10 °C every 15 minutes, until reaching a final temperature of 110 °C.

For the adhesion test, we prepared three smaller samples, each measuring 1 cm by 1 cm, cut from the larger silver-coated PET piece. The adhesion strength between the silver layer

and the PET substrate was evaluated using a pull-off test. In this setup, rigid substrates were attached to both sides of the samples: one side to the PET and the other to the silver film, using superglue for a secure attachment (Figure S5(a)). The pull-off test was conducted at a steady rate of 0.1 mm/min. The stress-displacement data obtained from the pull-off tests (Figure S5(b)) indicated an average adhesion strength of 2.7 MPa, with a standard error of 0.2 MPa, based on three separate measurements. Notably, post-test examination of the interface (Figure S5(c)) revealed that the silver coating remained intact and undamaged, suggesting that the actual adhesion strength between the silver and PET might be higher than the measured value. This conclusion is further supported by the fact that the adhesion between the silver and PET was observed to be stronger than the adhesion between the PET and the glue, limiting our ability to precisely determine the ultimate adhesion strength of the silver-PET combination.

References

(1) Liu, P.; Huang, B.; Peng, L.; Liu, L.; Gao, Q.; Wang, Y. A crack templated copper network film as a transparent conductive film and its application in organic light-emitting diode. *Scientific Reports* **2022**, *12*, 20494.

(2) Cui, M.; Zhang, X.; Rong, Q.; Nian, L.; Shui, L.; Zhou, G.; Li, N. High conductivity and transparency metal network fabricated by acrylic colloidal self-cracking template for flexible thermochromic device. *Organic Electronics* **2020**, *83*, 105763.

(3) Tran, V. V.; Nguyen, D. D.; Nguyen, A. T.; Hofmann, M.; Hsieh, Y.-P.; Kan, H.-C.; Hsu, C.-C. Electromagnetic Interference Shielding by Transparent Graphene/Nickel Mesh Films. *ACS Applied Nano Materials* **2020**, *3*, 7474–7481.

(4) Melnychenko, A. M.; Kudrawiec, R. Crack-Templated Wire-Like Semitransparent Electrodes with Unique Irregular Patterns. *ACS Omega* **2022**, *7*, 39181–39186.

(5) Han, B.; Pei, K.; Huang, Y.; Zhang, X.; Rong, Q.; Lin, Q.; Guo, Y.; Sun, T.; Guo, C.; Carnahan, D.; Giersig, M.; Wang, Y.; Gao, J.; Ren, Z.; Kempa, K. Uniform Self-Forming Metallic Network as a High-Performance Transparent Conductive Electrode. *Advanced Materials* **2014**, *26*, 873–877.

(6) Rao, K. D. M.; Gupta, R.; Kulkarni, G. U. Fabrication of Large Area, High-Performance, Transparent Conducting Electrodes Using a Spontaneously Formed Crackle Network as Template. *Advanced Materials Interfaces* **2014**, *1*, 1400090.

(7) Xian, Z.; Han, B.; Li, S.; Yang, C.; Wu, S.; Lu, X.; Gao, X.; Zeng, M.; Wang, Q.; Bai, P.; Naughton, M. J.; Zhou, G.; Liu, J.-M.; Kempa, K.; Gao, J. A Practical ITO Replacement Strategy: Sputtering-Free Processing of a Metallic Nanonetwork. *Advanced Materials Technologies* **2017**, *2*, 1700061.

(8) Gupta, R.; Rao, K. D. M.; Srivastava, K.; Kumar, A.; Kiruthika, S.; Kulkarni, G. U. Spray Coating of Crack Templates for the Fabrication of Transparent Conductors and Heaters on Flat and Curved Surfaces. *ACS Applied Materials & Interfaces* **2014**, *6*, 13688–13696.

(9) Rao, K. D. M.; Hunger, C.; Gupta, R.; Kulkarni, G. U.; Thelakkat, M. A cracked polymer templated metal network as a transparent conducting electrode for ITO-free organic solar cells. *Phys. Chem. Chem. Phys.* **2014**, *16*, 15107–15110.

(10) Guo, C. F.; Sun, T.; Liu, Q.; Suo, Z.; Ren, Z. Highly Stretchable and Transparent Nanomesh Electrodes Made by Grain Boundary Lithography. *Nat Commun* **2014**, *5*.

(11) Kang, S.; Arepalli, V. K.; Yang, E.; Lee, S.; Wi, J.-S.; Yun, J. H.; Song, S.; Kim, K.; Eo, Y.-J.; Cho, J.-S.; Gwak, J.; Chung, C.-H. High Performance and Flexible Electrodeposited Silver Mesh Transparent Conducting Electrodes Based on a Self-Cracking Template. *Electronic Materials Letters* **2022**, *18*, 440–446.

(12) Muzzillo, C. P.; Reese, M. O.; Lee, C.; Xiong, G. Cracked Film Lithography with CuGaO $_x$ Buffers for Bifacial CdTe Photovoltaics. *Small* **2023**, *19*, 2301939.

(13) Voronin, A. et al. Original concept of cracked template with controlled peeling of the cells perimeter for high performance transparent EMI shielding films. *Surfaces and Interfaces* **2023**, *38*, 102793.

(14) Voronin, A. S.; Fadeev, Y. V.; Govorun, I. V.; Podshivalov, I. V.; Simunin, M. M.; Tambasov, I. A.; Karpova, D. V.; Smolyarova, T. E.; Lukyanenko, A. V.; Karacharov, A. A.; Nemtsev, I. V.; Khartov, S. V. Cu–Ag and Ni–Ag Meshes Based on Cracked Template as Efficient Transparent Electromagnetic Shielding Coating with Excellent Mechanical Performance. *Journal of Materials Science* **2021**, *56*, 14741–14762.

(15) Yuan, C.; Huang, J.; Dong, Y.; Huang, X.; Lu, Y.; Li, J.; Tian, T.; Liu, W.; Song, W. Record-High Transparent Electromagnetic Interference Shielding Achieved by Simultaneous Microwave Fabry–Pérot Interference and Optical Antireflection. *ACS Applied Materials & Interfaces* **2020**, *12*, 26659–26669, PMID: 32422036.

(16) Wang, H.; Ji, C.; Zhang, C.; Zhang, Y.; Zhang, Z.; Lu, Z.; Tan, J.; Guo, L. J. Highly Transparent and Broadband Electromagnetic Interference Shielding Based on Ultrathin Doped Ag and Conducting Oxides Hybrid Film Structures. *ACS Applied Materials & Interfaces* **2019**, *11*, 11782–11791.

(17) Walia, S.; Singh, A. K.; Rao, V. S. G.; Bose, S.; Kulkarni, G. U. Metal Mesh-Based Transparent Electrodes as High-Performance EMI Shields. *Bulletin of Materials Science* **2020**, *43*, 187.

(18) Liao, D.; Zheng, Y.; Ma, X.; Fu, Y. Honeycomb-ring hybrid random mesh design with electromagnetic interference (EMI) shielding for low stray light. *Optics Express* **2023**, *31*, 32200.

(19) Kim, M.-H.; Joh, H.; Hong, S.-H.; Oh, S. J. Coupled Ag nanocrystal-based transparent mesh electrodes for transparent and flexible electro-magnetic interference shielding films. *Current Applied Physics* **2019**, *19*, 8–13.

(20) Lei, Q.; Luo, Z.; Zheng, X.; Lu, N.; Zhang, Y.; Huang, J.; Yang, L.; Gao, S.; Liang, Y.; He, S. Broadband Transparent and Flexible Silver Mesh for Efficient Electromagnetic Interference Shielding and High-Quality Free-Space Optical Communication. *Optical Materials Express* **2023**, *13*, 469–483, Publisher: Optica Publishing Group.

(21) Li, H.; Zhang, Y.; Tai, Y.; Zhu, X.; Qi, X.; Zhou, L.; Li, Z.; Lan, H. Flexible transparent electromagnetic interference shielding films with silver mesh fabricated using electric-field-driven microscale 3D printing. *Optics & Laser Technology* **2022**, *148*, 107717.

(22) Voronin, A. S. et al. Low Cost Embedded Copper Mesh Based on Cracked Template for Highly Durability Transparent EMI Shielding Films. *Materials* **2022**, *15*, 1449.

(23) Chung, S.-i.; Kim, P. K.; Ha, T.-g. High-performance transparent electromagnetic interference shielding film based on metal meshes. *Journal of Micromechanics and Microengineering* **2023**, *33*, 035002.

(24) Jiang, Z.-y.; Huang, W.; Chen, L.-s.; Liu, Y.-h. Ultrathin, Lightweight, and Freestanding Metallic Mesh for Transparent Electromagnetic Interference Shielding. *Optics Express* **2019**, *27*, 24194–24206, Publisher: Optica Publishing Group.

(25) Li, M.; Zarei, M.; Mohammadi, K.; Leu, P. W. Silver Meshes for Record-Performance Transparent Electromagnetic Interference Shielding. *ACS Applied Materials & Interfaces* **2023**,

(26) Ma, L.; Lu, Z.; Tan, J.; Liu, J.; Ding, X.; Black, N.; Li, T.; Gallop, J.; Hao, L. Transparent Conducting Graphene Hybrid Films to Improve Electromagnetic Interference (EMI) Shielding Performance of Graphene. *ACS Applied Materials & Interfaces* **2017**, *9*, 34221–34229, PMID: 28892351.

(27) Wang, H.; Lu, Z.; Liu, Y.; Tan, J.; Ma, L.; Lin, S. Double-Layer Interlaced Nested Multi-Ring Array Metallic Mesh for High-Performance Transparent Electromagnetic Interference Shielding. *Opt. Lett.* **2017**, *42*, 1620–1623.

(28) Liang, Z.; Zhao, Z.; Pu, M.; Luo, J.; Xie, X.; Wang, Y.; Guo, Y.; Ma, X.; Luo, X. Metallic nanomesh for high-performance transparent electromagnetic shielding. *Optical Materials Express* **2020**, *10*, 796.

(29) Liang, Y.; Huang, X.; Wen, K.; Wu, Z.; Yao, L.; Pan, J.; Liu, W.; Liu, P. Metal Mesh-Based Infrared Transparent EMI Shielding Window with Balanced Shielding Properties over a Wide Frequency Spectrum. *Applied Sciences* **2023**, *13*, 4846.

(30) Han, Y.; Zhong, H.; Liu, N.; Liu, Y.; Lin, J.; Jin, P. In Situ Surface Oxidized Copper Mesh Electrodes for High-Performance Transparent Electrical Heating and Electromagnetic Interference Shielding. *Advanced Electronic Materials* **2018**, *4*, 1800156.

(31) Shen, S.; Chen, S.-Y.; Zhang, D.-Y.; Liu, Y.-H. High-performance composite Ag-Ni mesh based flexible transparent conductive film as multifunctional devices. *Optics Express* **2018**, *26*, 27545.

(32) Han, Y.; Liu, Y.; Han, L.; Lin, J.; Jin, P. High-performance hierarchical graphene/metal-mesh film for optically transparent electromagnetic interference shielding. *Carbon* **2017**, *115*, 34–42.

(33) Jiang, Z.; Zhao, S.; Chen, L.; Liu, Y.-h. Freestanding "core-shell" AgNWs/metallic hybrid mesh electrodes for a highly efficient transparent electromagnetic interference shielding film. *Optics Express* **2021**, *29*, 18760.

(34) Lu, Z.; Ma, L.; Tan, J.; Wang, H.; Ding, X. Graphene, microscale metallic mesh, and transparent dielectric hybrid structure for excellent transparent electromagnetic interference shielding and absorbing. *2D Materials* **2017**, *4*, 025021.

(35) Han, Y.; Lin, J.; Liu, Y.; Fu, H.; Ma, Y.; Jin, P.; Tan, J. Crackle template based metallic mesh with highly homogeneous light transmission for high-performance transparent EMI shielding. *Scientific Reports* **2016**, *6*, 25601.

(36) Yang, Z.; Hao, Q.; Zhang, S.; Sun, X.; Tian, W.; Liu, F. Multispectral transparent electromagnetic-wave-absorbing optical window technology based on a random grid. *Optics Express* **2023**, *31*, 26355.
